# A Microfluidic Approach for Synthesis of Silver Nanoparticles as a Potential Antimicrobial Agent in Alginate–Hyaluronic Acid-Based Wound Dressings

**DOI:** 10.3390/ijms241411466

**Published:** 2023-07-14

**Authors:** Alexandra Cătălina Bîrcă, Oana Gherasim, Adelina-Gabriela Niculescu, Alexandru Mihai Grumezescu, Ionela Andreea Neacșu, Cristina Chircov, Bogdan Ștefan Vasile, Ovidiu Cristian Oprea, Ecaterina Andronescu, Miruna Silvia Stan, Carmen Curuțiu, Lia Mara Dițu, Alina Maria Holban

**Affiliations:** 1 Department of Science and Engineering of Oxide Materials and Nanomaterials, Politehnica University of Bucharest, 011061 Bucharest, Romania; 2 Lasers Department, National Institute for Lasers, Plasma and Radiation Physics, 409 Atomistilor Street, 077125 Magurele, Romania; 3 Research Institute of the University of Bucharest—ICUB, University of Bucharest, 050657 Bucharest, Romania; 4 Academy of Romanian Scientists, Ilfov No. 3, 050044 Bucharest, Romania; 5 Department of Inorganic Chemistry, Physical Chemistry and Electrochemistry, University Politehnica of Bucharest, 1-7 Polizu St., 011061 Bucharest, Romania; 6 Department of Microbiology and Immunology, Faculty of Biology, University of Bucharest, 077206 Bucharest, Romania

**Keywords:** microfluidic platform, silver nanoparticles, alginate-hyaluronic acid

## Abstract

The recognized antimicrobial activity of silver nanoparticles is a well-studied property, especially when designing and developing biomaterials with medical applications. As biological activity is closely related to the physicochemical characteristics of a material, aspects such as particle morphology and dimension should be considered. Microfluidic systems in continuous flow represent a promising method to control the size, shape, and size distribution of synthesized nanoparticles. Moreover, using microfluidics widens the synthesis options by creating and controlling parameters that are otherwise difficult to maintain in conventional batch procedures. This study used a microfluidic platform with a cross-shape design as an innovative method for synthesizing silver nanoparticles and varied the precursor concentration and the purging speed as experimental parameters. The compositional and microstructural characterization of the obtained samples was carried out by X-ray diffraction (XRD), scanning electron microscopy (SEM), transmission electron microscopy (TEM), and dynamic light scattering (DLS). Four formulations of alginate-based hydrogels with the addition of hyaluronic acid and silver nanoparticles were obtained to highlight the antimicrobial activity of silver nanoparticles and the efficiency of such a composite in wound treatment. The porous structure, swelling capacity, and biological properties were evaluated through physicochemical analysis (FT-IR and SEM) and through contact with prokaryotic and eukaryotic cells. The results of the physicochemical and biological investigations revealed desirable characteristics for performant wound dressings (i.e., biocompatibility, appropriate porous structure, swelling rate, and degradation rate, ability to inhibit biofilm formation, and cell growth stimulation capacity), and the obtained materials are thus recommended for treating chronic and infected wounds.

## 1. Introduction

Numerous scientific papers have demonstrated silver’s attractive and optimal properties for utilization in biomedical applications, such as its small size, high surface area-to-volume ratio, biocompatibility, and notable effects against many bacterial strains and viruses [1,2,3,4,5,6,7]. This antimicrobial agent possesses a comprehensive microorganism spectrum that is active on Gram-positive and Gram-negative strains. Usually, smaller particles exhibit a more powerful antibacterial effect when compared with bigger particles [8,9,10,11]. This may be explained by the fact that the atoms are shown on the surface in an increased concentration, which allows for improved reactivity and biocidal action. As a result, numerous medical applications of silver nanoparticles have been established and demonstrated, including drug delivery, disease diagnosis, chronic wound treatment, and dermato-cosmetics, as well as in coatings for medical devices, surgical instruments, masks, implants, and others [12,13,14,15,16,17,18,19].

In this sense, synthesis methods for silver nanoparticles can vary considerably in order to finally achieve tailored characteristics [20,21,22,23]. Microfluidic technology offers advantageous results to reduce or eliminate possible human factors that do not always allow for reproducibility and to obtain some of the final desired properties [14,24,25,26].

A microfluidic system is a miniaturized experimental set-up, also called lab-on-a-chip, which generally consists of a platform conventionally obtained from plastic materials for manipulating small amounts of fluids. The material is specifically chosen for the platform because of its transparency, chemical compatibility with the precursors, mechanical properties, and biocompatibility, which are important factors to consider. Hence, a deep understanding of the entire system is needed to ensure that the reaction conditions are controlled, reproducible, and able to produce materials that cover the requirements in terms of size and morphology [25,27,28,29,30]. 

Silver nanoparticle synthesis through a microfluidic platform represents a facile, low-cost, reproducible method to develop the fundamental characteristics required for advanced applications. Within the next few years, microfluidic silver synthesis will likely become one of the most important and extended techniques, creating considerable interest in the biomedical field and beyond [24,26,31,32,33].

One of the most promising applications of silver nanoparticles in the biomedical field is its utilization as an antimicrobial agent in wound dressings. As wounds are open injuries that may be affected by microorganisms, which can lead to infection, using silver nanoparticles is a suitable method to avoid contamination of the affected skin [34,35,36,37,38,39]. According to the literature, a strong and demonstrated concept regarding ideal antimicrobial wound dressings is based on the hydrogel-type dressing. Therefore, hydrogel dressings are classed highly as to have the required properties for ideal wound dressing materials. The use of hydrogels in wound management supports many activities necessary for improving the healing process, such as the capacity to adsorb the wound exudate, provide gas exchange, assure and maintain a moist environment, avoid permeability to microorganisms, biocompatibility, and biodegradability [40,41,42].

Regarding those listed previously, alginate has been described as a biopolymer that can reach and overcome the obstacles still faced by patients and medical personnel. Alginate dressings can manage the wound exudate by absorbing it in dry form, supporting a moist environment, and promoting skin regeneration while showing a hemostatic activity. The most common hemostatic alginate material is calcium alginate, which provides calcium ions at the level of skin injury through contact with sodium ions found in the blood via the ion exchange reaction [17,41,43,44,45,46,47,48,49]. Thus, wound healing can be enhanced by silver nanoparticle incorporation into alginate-based hydrogels.

In addition, it is well known that decreasing the size of silver particles leads to modifications in the electronic structure, improving the microorganism-ruining action. Moreover, the particle size property is related to the slow and controlled release and the oxidation mechanism of Ag^+^ in the body fluid conditions. Using silver nanoparticles with hydrophilic alginate-based hydrogels promotes a hydrophilic environment that encourages releasing nanoparticles from the polymeric matrix [34,50,51,52,53,54,55]. 

Hyaluronic acid (HA) belongs to the glycosaminoglycans family and is found in the extracellular matrix, and it is a natural and essential component of tissues, especially in epithelial and neural tissues. HA naturally supports cell proliferation and angiogenesis and is also involved in fetal development and improving tissue function. The characteristics of HA are of interest in biomedical applications because biocompatibility and biodegradability are categorized as highly important in tissue regeneration. The biological effects of HA are in line with the molecular weight. In addition, composite materials with HA used as a wound dressing seem to stimulate vascularization and angiogenetic capacity, which is an important factor in every stage of wound healing. Moreover, HA also has properties such as bacteriostatic activity (not bactericidal) on different planktonic cells, which have been proven to act against biofilm formation [50,56,57,58,59,60,61,62,63,64,65,66,67,68,69]. 

Therefore, combining the benefits of nano-silver, alginate, and hyaluronic acid is expected to bring about a synergistic activity in wound care. Evidence from the literature indicates that different composites based on these materials exhibit suitable physicomechanical properties for wound dressings (i.e., improved absorption, tensile, and gelation properties). Moreover, they constitute effective products for restoring skin homeostasis by treating infected tissues and stimulating wound-healing processes [50,64,70,71]. 

Considering the above, this study proposes using a cross-shape microfluidic platform for the on-chip synthesis of silver nanoparticles by reducing silver ions in the presence of sodium hydroxide and D-glucose at room temperature. The obtained nanoparticles were characterized to investigate the crystallinity and crystallite size, particle size, particle zeta potential, and hydrodynamic radius. Moreover, alginate-based hydrogels were obtained from silver nanoparticles and with hyaluronic acid with suitable characteristics for improved healing of the wounds. Alginate-based hydrogels were evaluated according to their morphostructure, pore size, swelling capacity, and degradation rate. Highlighting the application of the obtained materials in wound management, the antimicrobial and biocompatibility evaluation results are reliable for the targeted application. Thus, by presenting a novel synthesis route for antimicrobial composite hydrogels, this article contributes to developing wound dressings with controlled properties, improved physicochemical characteristics, and enhanced biological activities in a timely and effective manner. 

## 2. Results

### 2.1. Silver Nanoparticles Characterization

**Figure 1:**
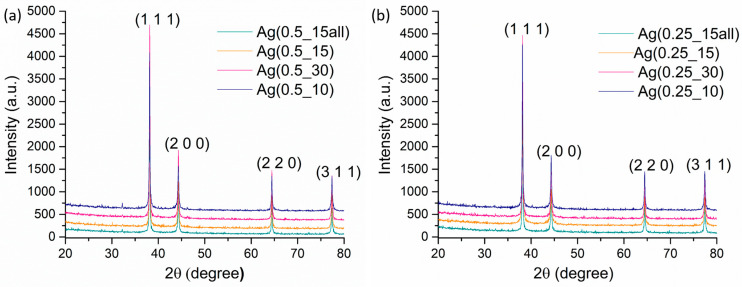
X-ray diffractogram performed for the series of samples with 0.5% silver nitrate: (**a**) Ag(0.5_15all), Ag(0.5_15), Ag(0.5_30), and Ag(0.5_10). X-ray diffractogram performed for the series of samples with 0.25% silver nitrate: (**b**) Ag(0.25_15all), Ag(0.25_15), Ag(0.25_30), and Ag(0.25_10)).

X-ray diffractograms were obtained for both concentration series, and the resulting patterns are shown in Figure 1a,b. Based on the PDF-ICDD database, metallic silver was identified as a single phase in all of the analyzed samples (reference code 04-014-0266), with a cubic crystallographic system and Fm-3m space group. The four main peaks identified correspond to (1 1 1), (2 0 0), (2 2 0), and (3 1 1) Miller indexes and their high intensity suggests a well-crystalized material.

To evaluate the crystallite size for each sample, we used the Debye–Scherrer formula:

$$D=\frac{0.9\;\lambda }{\beta \cos\theta }$$

where *D* is the calculated crystallite size, λ is the wavelength of the X-ray used by the equipment (0.1540 nm), β is represented by the full width at half maximum (FWHM), and θ is the diffraction angle. 

From the results observed in Table 1, we can conclude that the smaller average crystallite size was registered for the Ag(0.5_10) sample, where the channel speed for the silver nitrate was set at 10 RPM, and the channel speed for the reducing solution was set at 15 RPM. In this sense, the crystallite size decreased with increased organic solution flow. 

The smaller average crystallite size for the samples with 0.25% silver concentration (detailed in Table 2) was found for the Ag(0.25_15) sample, where the tube for silver nitrate was set at 15 RPM, and the tubes for the reduction solution were set at 30 RPM. Here, it can also be concluded that the crystallite size decreased with the increase in organic solution flow. 

For both series of samples, the samples with the greatest amount of silver nitrate solution (sample Ag(0.5_30) and Ag(0.25_30) had the greatest crystallite size results. However, a decrease in the flow of the reduction solution led to an increase in the crystallite size of the samples. In addition, the results of the samples with 0.25% silver nitrate concentration presented lower values compared with the samples with 0.5% silver nitrate concentration. 

SEM micrographs show the influence of the synthesis parameters in the final morphology of the silver powders. The morphology of the particles was quasi-spherical, and the dimensions varied in the nanometric range. 

**Figure 2:**
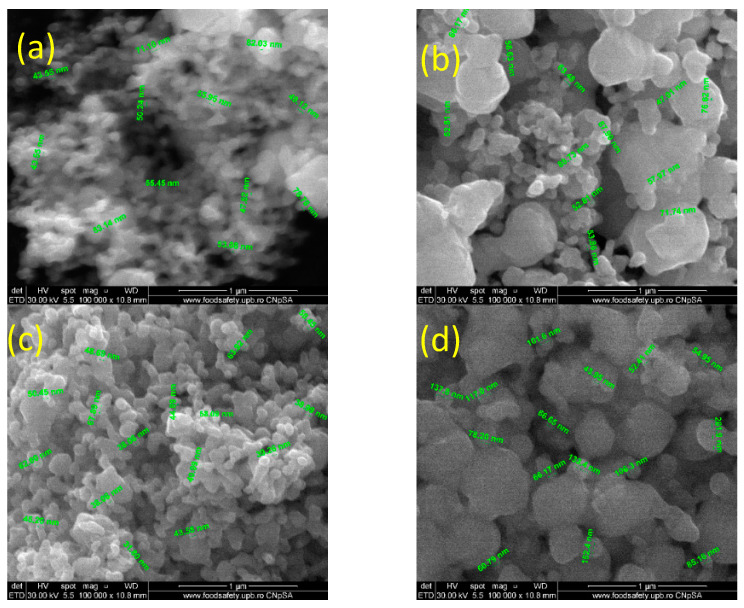
SEM micrographs obtained for the first series of samples with 0.5% silver nitrate: (**a**) Ag(0.5_15all), (**b**) Ag(0.5_15), (**c**) Ag(0.5_30), and (**d**) Ag(0.5_10).

All of the samples from the 0.5 wt.% series exhibited the formation of agglomerates correlated with the small nanometric dimensions of the particles (Figure 2). In most cases, a dimensional non-uniformity of the particles was observed, with the only homogenous sample being the Ag(0.5_30) sample. This suggests that a higher flow rate for the silver nitrate solution was needed to obtain the optimal morphological and dimensional characteristics.

**Figure 3:**
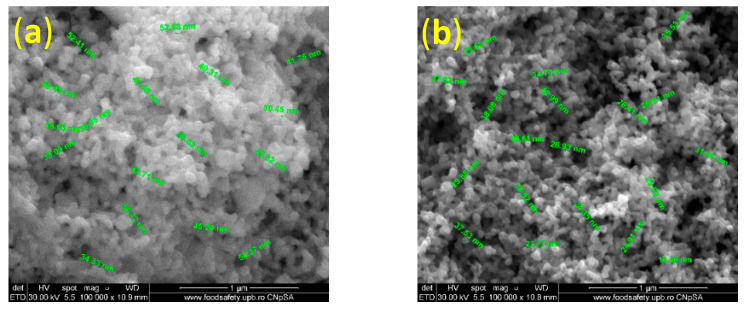

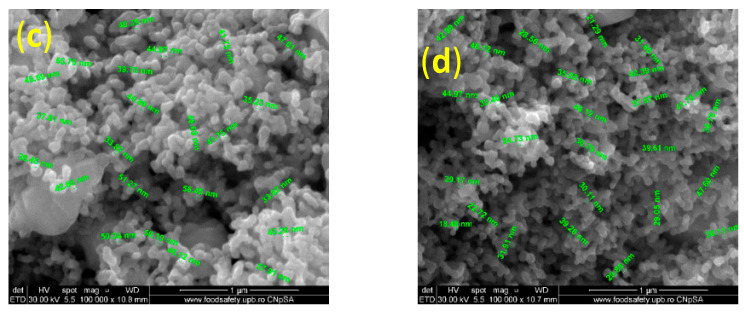
SEM micrographs obtained for the second series of samples, with 0.25% silver nitrate: (**a**) Ag(0.25_15all), (**b**) Ag(0.25_15), (**c**) Ag(0.25_30), and (**d**) Ag(0.25_10).

Below are the SEM micrographs recorded for series 2 with 0.25% silver nitrate (Figure 3). Following the synthesis parameters, all four of the obtained samples were analyzed to observe their characteristics. 

For series 2, the results, at first glance, were more promising compared with series 1. Notably, introducing a lower percentage of silver precursors in the microfluidic system avoided the formation of agglomerates, even though the nanometric size was maintained. However, in line with synthesizing small nanoparticles with a uniform size distribution by microfluidic systems, it can be concluded that the experimental results found clear support for using a small percentage of silver precursors. The sample with optimal results for the dimensional and morphological characteristics from series 2 was Ag(0.25_15). In this case, the silver nitrate input was set at 15 RPM, and the reduction solution inputs were set at 30 RPM. In contrast with the results from series 1, a slow flow of the inorganic precursor was needed to reach the optimal characteristics. 

Bright-field and high-resolution TEM images were obtained for both series of silver samples. In addition, correlated with the XRD results, a selected area electron diffraction (SAED) module was used to identify the atom rings of the samples. 

**Figure 4:**
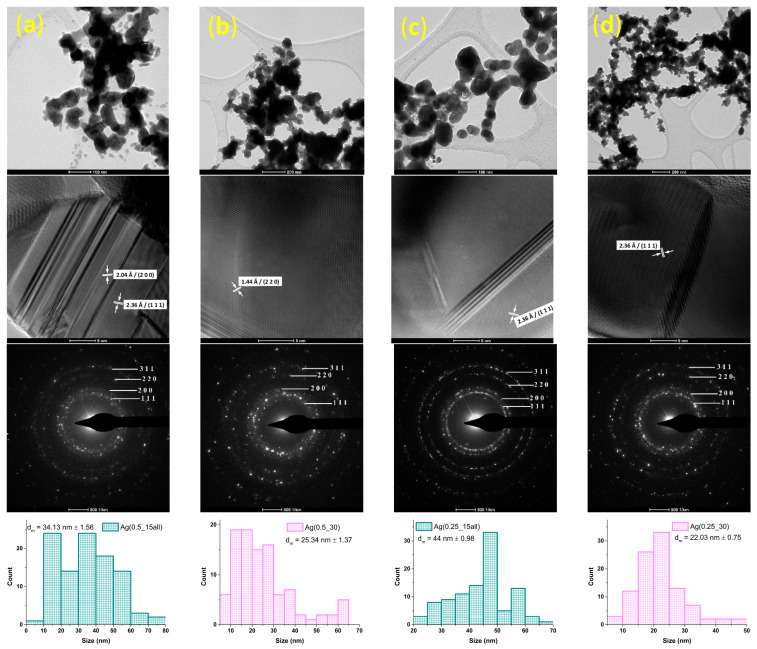
Bright-field and high-resolution TEM, SAED, and size distribution of nanoparticles: (**a**) Ag(0.5_15al), (**b**) Ag(0.5_30), (**c**) Ag(0.25_15all, and (**d**) Ag(0.25_30).

TEM analysis revealed in Figure 4, confirmed the spherical and pseudo-spherical morphology of the silver nanoparticles with a tendency towards agglomeration. By comparing the TEM bright-field micrographs obtained for samples with 0.5% silver nitrate, the samples’ morphology, and size were quite different than for the 0.25% silver nitrate samples, where the size of the particles was smaller, and the morphology was more spherical. An interplanar distance of 2.36 Å was observed in the HR-TEM images, corresponding to the (111) planes, and confirmed the Fm-3m cubic structure of the silver nanoparticles. The particle size was not equal to the crystallite size, calculated from XRD data, which confirms that the particles were polycrystalline and the particles were organized from 2 or 3 crystallites. From the SAED patterns, it was confirmed that the single phase obtained for all the samples regarding the spotted rings matched (311), (220), (200), and (111) silver lattice planes. 

**Figure 5:**
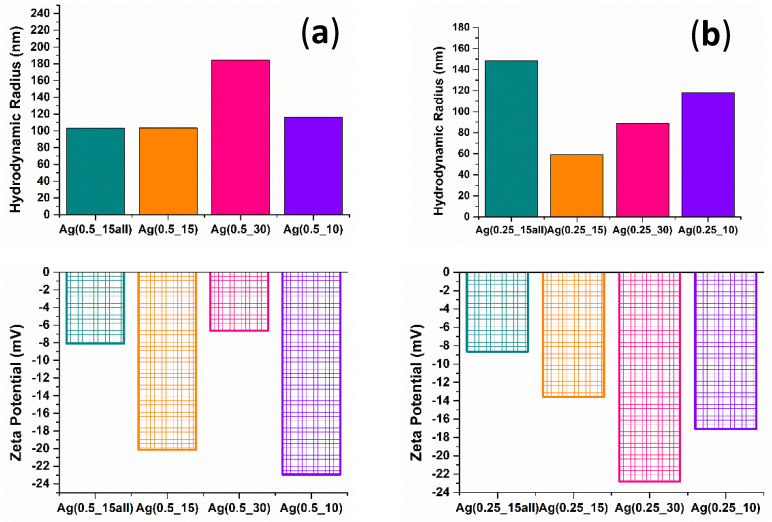
Hydrodynamic radius and zeta potential of silver nanoparticles measured using the DLS technique for the first series of samples with 0.5% silver nitrate: (**a**) Ag(0.5_15all), Ag(0.5_15), Ag(0.5_30), and Ag(0.5_10). For the second series of samples, with 0.25% silver nitrate: (**b**) Ag(0.25_15all), Ag(0.25_15), Ag(0.25_30), and Ag(0.25_10).

A dynamic light scattering technique was used to determine the hydrodynamic diameter distribution of silver nanoparticles in the suspension and their zeta potential. The results are presented in Figure 5. 

The DLS results show that in the case of the series 1 pattern, the smallest hydrodynamic radius corresponded to the Ag(0.5_15) sample, and the biggest corresponded to Ag(0.5_30). In contrast with the TEM results, where the Ag(0.5_30) sample exhibited a smaller particle size from the bright-field images and histogram, the possible explanation is that during DLS analysis, the small particles could have been hidden by the agglomerates, and it was difficult to measure the particles individually. The zeta potential value for the Ag(0.5_15) sample was −20.11 mV, indicating moderate suspension stability compared with −6.66 mV, where the instability of the Ag(0.5_30) sample was observed. For series 2, the DLS pattern revealed some expected results. The hydrodynamic radius for Ag(0.25_30) was lower than Ag(0.25_15all), and these results correspond to the TEM images and size distribution from the histogram. In addition, the smallest hydrodynamic radius was for Ag(0.25_15), having 58.97 nm. Referring to the zeta potential results, the best values and stability in the suspension were noticed for the Ag(0.25_30) sample, which was correlated with the TEM analysis data. 

### 2.2. Hydrogels Characterization

**Figure 6:**
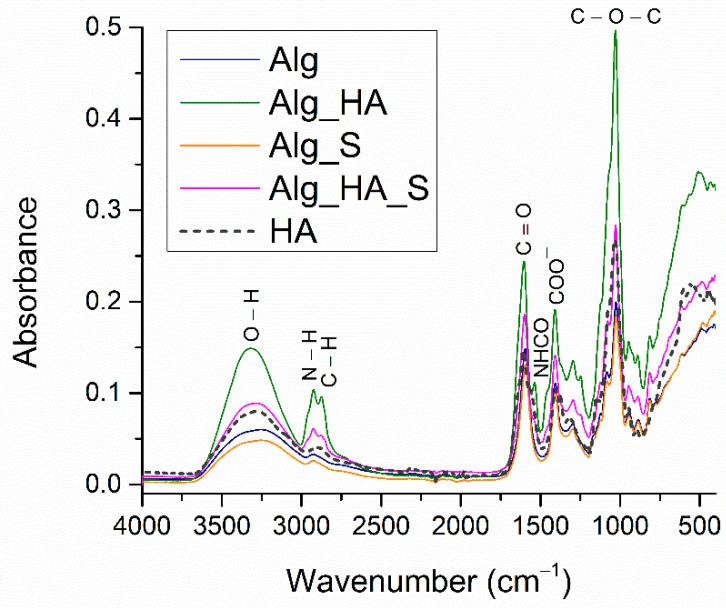
FT-IR spectra for the Alg, Alg_HA, Alg_S, and Alg_HA_S samples and HA.

FTIR spectra were recorded for all hydrogels in order to confirm the functional groups found in the obtained samples (Alg, Alg_HA, Alg_S, and Alg_HA_S). Figure 6 shows the results for each hydrogel with the fingerprint of the hydrophilic feature of the alginate by presenting a broad vibration band between 3100–3400 cm^−1^, characteristic of OH stretching.

The absorption band observed around 1020 cm^−1^ was correlated with the C-O-C functional group regarding the alginate saccharide structure. COO^-^ was also evidenced at 1409 cm^−1^, also characteristic of alginate. Hyaluronic acid absorption bands confirmed the amide group found in its chemical structure, which was observed due to the N-H stretching at 2924 cm^−1^. In addition, an indicative wavenumber was 1603 cm^−1^, showing the asymmetric stretching for the carbonyl group from a glucuronic unit observed in the Alg_HA spectrum [72,73,74]. The most intense OH vibration bands were for Alg_HA and Alg_HA_S, regarding the hydrophilic feature of hyaluronic acid, an important aspect of wound healing. The FTIR spectrum for the hydrogels with silver nanoparticles (Alg_S and Alg_HA_S) exhibited only shifts in the absorption bands. 

**Figure 7:**
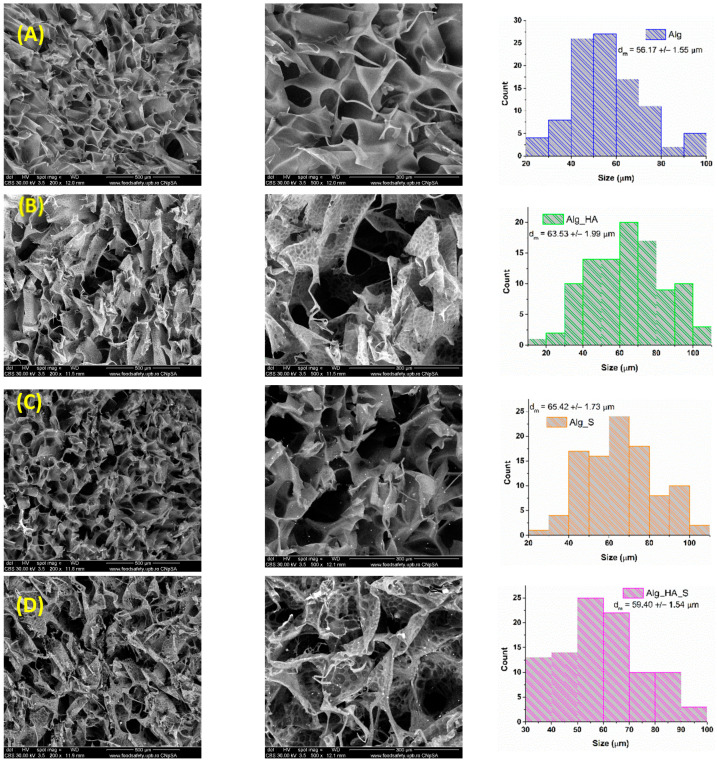
SEM micrographs for Alg (**A**), Alg_HA (**B**), Alg_S (**C**), and Alg_HA_S (**D**), and the pore size distribution.

SEM analysis was performed to evaluate the morphology and pore size of the obtained hydrogels. The resulting micrographs are presented in Figure 7 at two magnifications (200× and 500×). The morphology of the hydrogels was in accordance with the requirements for an ideal dressing due to the porous structure found in all of the hydrogels. A difference between the alginate and alginate–hyaluronic acid hydrogels is represented by the organization of the pores; namely, the alginate hydrogels had round pores (Figure 7A,C), but after the addition of hyaluronic acid, the pores became more elongated (Figure 7B,D). Referring to the pore size distribution, presented as a histrogram in Figure 7 for each hydrogel, it can be seen that the alginate with hyaluronic acid incorporation led to a larger dimension of the pores. The biggest pore size value (approximately 99 µm) was calculated for Alg_HA_S, compared with Alg, where the smallest pore size was found. The uniform distribution of silver nanoparticles (Ag0.25_15) is evidenced in Figure 7C for Alg_S and (Figure 7D) for Alg_HA_S. In addition, after the addition of hyaluronic acid, silver nanoparticles were distributed more evenly in the soft-like morphology of the pores. In this sense, adding hyaluronic acid to the alginate hydrogels promoted an ideal dressing by changing the smooth feature, the pore shape, and the homogeneity of silver nanoparticle distribution within the hydrogel.

**Figure 8:**
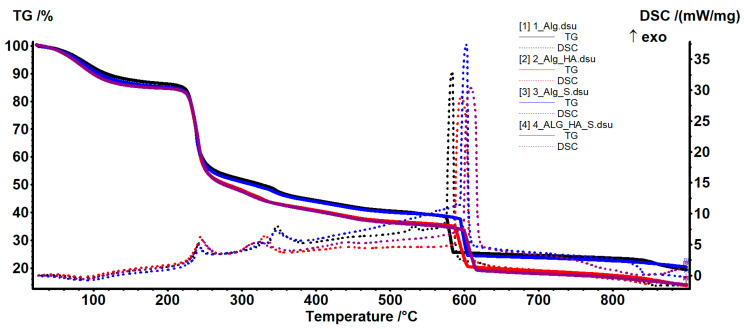
Thermogravimetry/differential scanning calorimetry (TG-DSC) results for the Alg, Alg_HA, Alg_S, and Alg_HA_S hydrogels.

From the thermal analysis (TG-DSC) results shown in Figure 8, it is clear that the samples had a similar thermal behavior for samples grouped with (Alg_HA and Alg_HA_S) and without hyaluronic acid (Alg and Alg_S). 

The samples without hyaluronic acid lost the residual solvent molecules and underwent dehydration up to 210 °C, and the process was accompanied by an endothermic effect on the DSC curve, with a minimum at 88.2 °C for Alg and 89.1 °C for Alg_S. The first decomposition process occurred between 210–280 °C when the polymer backbone was fragmented, and some oxidation reactions took place as indicated by the exothermic peak at 241.2 °C for Alg and 242 °C for Alg_S. The Alg-recorded mass loss was 32.54% and for Alg_S was 32.33%. The degradative oxidation continued in the interval of 280–400 °C when a mass loss of 9.04% for Alg and 8.61% for Alg_S were recorded, together with the exothermic peak from 347.2 °C and 346.0 °C, respectively. After 400 °C, the samples were fully oxidized, and the carbonaceous mass was burned away, while the residual mass was recorded as 19.37% for the Alg sample and 20.29% for the Alg_S, respectively. The differences between the samples with hyaluronic acid (HA) are indicated by the data in Table 1. The presence of HA induced a larger mass loss after 210 °C (34.44% for Alg_HA and 34.80% for Alg_HA_S). The residual mass was smaller, considering the concentration of hyaluronic acid solutions. The silver content calculated for the Alg_S sample was 1.27%, and for Alg_HA_S, it was 0.36%, as specified in Table 3. With the concentration of the alginate solution being 5% and the hyaluronic acid solution being 3%, and the polymer volumes being equal for each sample, it is expected that the residual mass in the samples with hyaluronic acid will be lower, and hence the difference in silver content between Alg_S and Alg_HA_S.

**Figure 9:**
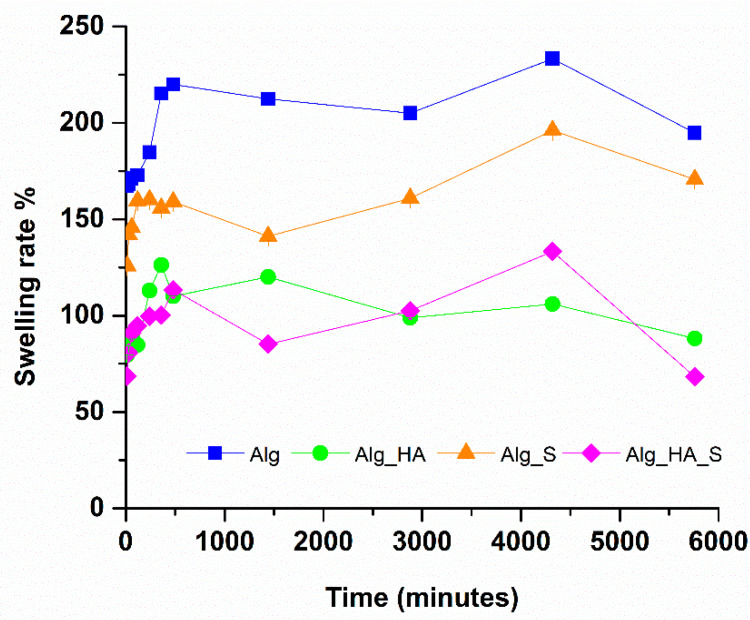
Time-dependent swelling rate evaluation for Alg, Alg_HA, Alg_S, and Alg_HA_S.

The swelling rate evaluation showed the estimative capacity of the hydrogels to swell in contact with a prepared simulated body fluid. All hydrogels were tested to identify the most suitable material to be used for absorbing the exudate from the injured tissue. The results are represented in Figure 9.

**Figure 10:**
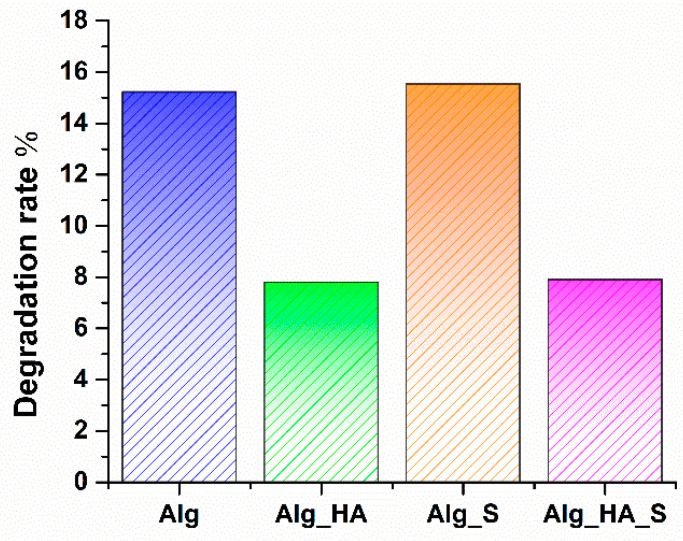
Degradation rate evaluation of Alg, Alg_HA, Alg_S, and Alg_HA_S after 96 h.

All hydrogels recorded a swelling degree at every weighted time. It is observed that Alg and Alg_S had the greatest increase in swelling rate. However, Alg_HA and Alg_HA_S reached 70% maximum swelling in the first 15 min, and then the swelling rate increased, averaging 99% for Alg_HA and 94% for Alg_HA_S, respectively. The values were approximately similar at 8–72 h, but after 72 h (4320 min), a decrease in swelling capacity was observed. This may be correlated with the degradation rate results (Figure 10), where all hydrogels recorded a decrease in the material mass, which was estimated as 15% for both Alg and Alg_S samples and 7% for both samples with hyaluronic acid (Alg_HA and Alg_HA_S) after 96 h. 

Another difference was observed between alginate and alginate–hyaluronic degradation; namely, after 96 h, the hydrogels with hyaluronic acid had a lower degradation rate compared with the alginate hydrogels. According to the literature data, the biodegradability of materials that are used in wound healing and skin regeneration is an important and required feature. 

**Figure 11:**
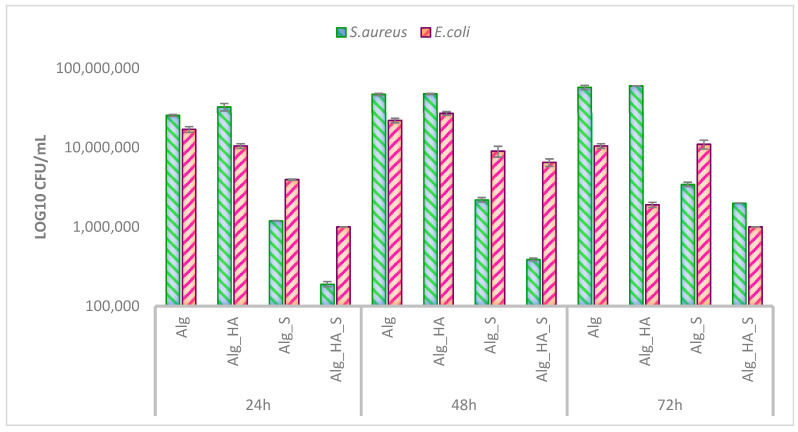
Graphic representation of the values (log10 CFU/mL) obtained for expressing biofilm modulation against Gram-positive and Gram-negative bacterial strains on hydrogels at 24, 48, and 72 h.

A major issue in chronic wounds is the infection of the affected site, which is often further represented in biofilm growth. It is well known that if bacteria colonization translates into thick biofilm development, wound healing would be difficult to manage because the formed biofilm that is formed is resistant to antibiotics or any usual biocide. To clarify if the obtained materials are efficient at combating the bacterial biofilm, it is necessary to evaluate the growth dynamic at different times (24, 48, and 72 h). In this sense, the hydrogels were subsequently subjected to in vitro tests according to the mentioned protocol to evaluate the anti-biofilm potential of the developed wound dressings. The evaluation was made on *Staphylococcus aureus* (*S. aureus*) and *Escherichia coli* (*E. coli*) strains (Figure 11) by comparison with Alg and Alg_HA hydrogels as a control to highlight the activity of the silver nanoparticles present in the samples.

The resulting data suggest that biofilm formation is inhibited by silver nanoparticles containing hydrogels (Alg_S and Alg_HA_S). Comparing the antimicrobial activity of the hydrogels against *S. aureus* and *E. coli*, it was observed more in the diminishing biofilms produced by the Gram-positive strain. We found that after 24 h, hydrogels Alg_S and Alg_HA_S exhibited the most significant biofilm inhibition, and the activity was maintained even after 48 h. After 72 h, the antimicrobial agent still had efficiency for inhibiting the bacterial film, according to our results. Alg and Alg_HA hydrogels presented high values of CFU/mL in all of the analyzed times on the *S. aureus* strain.

On the other hand, a decrease in biofilm inhibition in *E. coli* was observed for all of the tested time points. Alg_S and Alg_HA_S had different reactions for inhibiting the Gram-positive and Gram-negative bacteria. For the hydrogels with silver nanoparticles, increased inhibition activity was observed against the *S. aureus* strain, which is often found to cause difficult wound infections. Moreover, the Alg_HA_S hydrogel exhibited decreased CFU/mL values for all of the times tested, compared with the Alg_S hydrogel. This may be possible due to the bacteriostatic effect of hyaluronic acid on microorganisms. According to our results, the bioactivity of Alg_S and Alg_HA_S was maintained for at least 72 h by inhibiting the bacterial biofilm (both Gram-positive and Gram-negative). 

**Figure 12:**
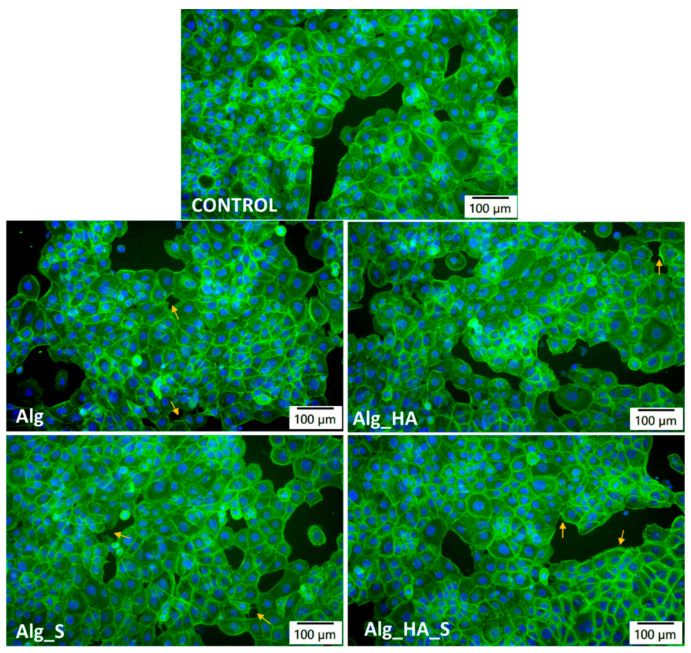
Alginate-based hydrogels (Alg, Alg_HA, Alg_S, and Alg_HA_S samples) after 24 h in the presence of HaCaT keratinocytes, as shown by the fluorescence staining of the actin filaments (green: F-actin labeled with phalloidin-FITC; blue: nuclei labeled with DAPI; arrows: actin-based protrusions, scale bar: 100 µm).

Considering the application of the obtained materials in our study, the interaction with specific skin cell lines was very beneficial for obtaining more information about the properties of the hydrogels. Biocompatibility, for instance, is an essential characteristic that should be assessed and included in the hydrogel checklist. To assess the cellular morphology in the presence of alginate-based hydrogels, the fluorescence staining of actin filaments in HaCaT keratinocytes was performed (Figure 12). Similar cell densities for all types of samples were observed compared with the control (cells without Alg-based samples) after 24 h of incubation. The microscopy images revealed cell-to-cell junctions being noticed in actin-based protrusions (Figure 12—arrows), such as lamellipodia and filopodia. These images confirmed the healthy morphology of the actin cytoskeleton in the presence of these hydrogels. 

**Figure 13:**
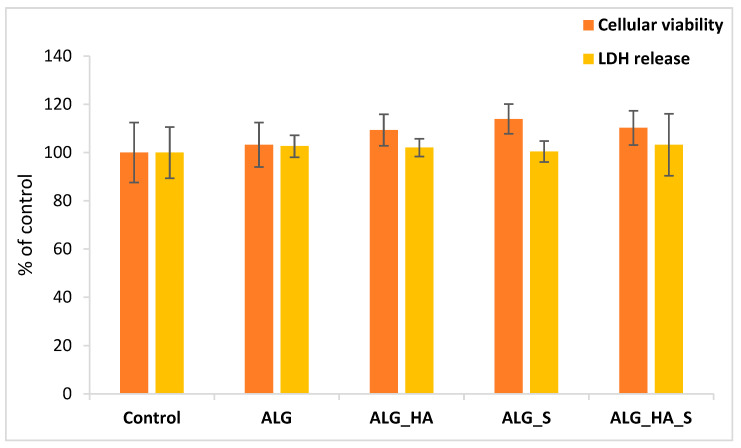
Alginate-based hydrogels (Alg, Alg_HA, Alg_S, and Alg_HA_S samples) proved good biocompatibility after 24 h in the presence of HaCaT keratinocytes, as shown by the MTT and LDH assays (results are presented as mean ± standard deviation of three independent experiments and represented as percentages of negative control (cells without hydrogels)).

In addition, the cellular viability results obtained by MTT assay (Figure 13) proved the hydrogels’ good biocompatibility, agreeing with the fluorescence microscopy. Regarding cell viability, it can be observed that, apart from the Alg sample, all the other three samples (Alg_HA, Alg_S, and Alg_HA_S) stimulated cell growth during the tested time of 24 h. This is very important for a hydrogel-type material because, from the first hours of contact with human keratinocytes, they have support in terms of their development. Moreover, the integrity of the cell membrane was maintained after 24 h of incubation, as measured by the levels of LDH release (Figure 13), which were almost the same for the control and alginate-based samples.

## 3. Discussion

Until recent years, the conventional synthesis methods for nanoparticles were precipitation, sol–gel, micro-emulsion, etc., where processes such as nucleation, growth, and agglomeration unavoidably happen simultaneously [75,76]. However, these conventional methods have several limitations, and the microfluidic synthesis method for nanoparticles has become a more interesting, efficient, and controllable way to obtain nanoparticles with improved properties suitable for biomedical applications. According to the literature data, the first experiment for obtaining silver nanoparticles was conducted through a continuous single-flow tubular microfluidic system, in which, through thermal reduction, nanoparticles were obtained within minutes [26,33]. 

Because of the final properties of nanoparticles reached through a microfluidic method, this study investigated the physicochemical characteristics of silver samples obtained by setting up the silver nitrate concentration and rotation per minute (RPM), conducted via a cross-shape microfluidic platform. The single silver phase was supported by XRD diffractograms through the identification of characteristic peaks with Miller indices of (111), (200), (220), and (311) for both series of samples, with crystallite size between 42–54 nm for series with 0.5% silver nitrate and 31–44 nm for series with 0.25% silver nitrate. Lei Xu et al. [77] showed that the crystallite size for microfluidic-synthesized Ag was 13.88 nm, using sodium borohydride as a reducing agent. However, the emphasis when evaluating the characteristics of microfluidic silver nanoparticles is based on the particle size from the SEM analysis. Our results revealed a difference between series with 0.5% and 0.25% silver nitrate. More specifically, the influence of silver precursor concentration on the size of the nanoparticles was one of the most important parameters [78,79]. Smaller dimensions were obtained for samples with a lower concentration of silver nitrate, as shown in the SEM results. Hong et al. used a microdroplet method to synthesize silver nanoparticles. The silver nanoparticle size results indicated that the size increased with the silver precursor concentration [80]. Sobczak-Kupiec et al. also demonstrated the influence of the silver nitrate concentration on the size of the silver nanoparticles [81]. In addition, the reducing agent was a critical parameter because a strong reducing agent such as NaBH_4_ may promote fast nuclei generation, which supports obtaining smaller nanoparticles compared with a weaker reducing agent [82]. The SEM micrographs in our study obtained values for nanoparticles sized between 38–100 nm for series 1 and between 18–50 nm for series 2, where the concentration of silver nitrate was decreased. Numerous research papers have aimed to obtain particles with smaller sizes (5–10 nm). Xu et al. [77] obtained particles through a microfluidic device with a mean diameter of 13.2 nm; then, by adding NH_4_OH as a complexant, the particle size decreased, leading to a mean diameter of 9.1 nm. Another experiment was done by adding ethylenediaminetetraacetic acid (EDTA) as a complexant, where the particles were 4.9 nm. Ng et al. [83] used a rotating packed bed reactor and produced silver nanoparticles with an average size of 20–25 nm by optimizing parameters such as the rotation per minute and reducing agent concentration. Lazarus et al. [84] synthesized silver nanoparticles with a diameter of 3.73 nm using a two-phase microfluidic droplet. Baber et al. [85] obtained sizes of silver nanoparticles in the range of 3.1–5.6 nm using a microfluidic coaxial flow reactor, and showed that by increasing the flow rates, the size increased too.

Depending on the final application of silver nanoparticles, the size is essential. Our study used these nanoparticles to produce a hydrogel with antimicrobial and regenerative properties, and thus cytotoxicity is a critical evaluation. Various papers have focused on demonstrating the importance of silver nanoparticle sizes in biomedical applications. Kim et al. [86] evaluated cell apoptosis for three sizes of silver nanoparticles, namely 10, 50, and 100 nm, in contact with osteoblastic MC3T3-E1 and pheochromocytoma PC12 cell lines. The evaluation was made using a flow cytometer, and it was revealed that compared with the MC3T3-E1 control (without silver nanoparticles), the cells treated with 10 µg/mL showed increased apoptosis: 5.05% (10 nm), 2.49% (50 nm), and 1.11% (100 nm). The results demonstrated the size-dependent activity of the silver nanoparticles, with apoptosis being observed the most in the smallest nanoparticles (10 nm). Apoptosis of smaller silver nanoparticles (between 5–20 nm) was observed by Li et al. in a study, compared with sizes of 50 nm that did not affect the viability of the cells [86]. Toxicity evaluation of size-dependent silver nanoparticles was performed on four cell lines and showed that the smaller sizes were more toxic than, the larger sizes (5 nm in contrast with 20 and 50 nm) [87]. Regarding these research papers, based on the size-dependent silver nanoparticles toxicity, our results referring to nanoparticle size (SEM and TEM) were suitable for proper use in biomedical applications. 

The hydrodynamic radius and zeta potential were measured for all of the silver nanoparticles obtained in this study. According to the presented results, the hydrodynamic radius for silver samples with a lower concentration of inorganic solution was smaller than for series 1 samples. The values for series 1 were in the range of 103–184 nm, with the highest radius (184.53 nm) for Ag(0.5_30) and the smallest (103.07) for Ag(0.5_15). In contrast, series 2 exhibited a more favorable outcome, having values for hydrodynamic radius between 58 and 148 nm. However, the lowest value was recorded for Ag(0.25_15), showing about 58 nm. The Ag(0.25_15) result was correlated with the SEM measurement for particle size. The zeta potential pointed out the negative charge and the stability of the samples. In a study performed by Magdalene et al. [88], the DLS results for silver nanoparticles obtained through a microfluidic green synthesis revealed a particle size close to 72 nm. Hassoun et al. synthesized silver nanoparticles using a microfluidic chip, and the result for the particle size was 50 nm, according to the DLS analysis. 

Eight samples of silver nanoparticles were synthesized through a cross-shape microfluidic platform using two silver nitrate concentrations and four different RPMs for the inputs of the reaction solutions. The results allowed for the identification of the optimal parameters for obtaining nanoparticles with the necessary properties to be used later in tissue engineering. Ag(0.25_15) presented favorable results to be used as an antimicrobial agent in hydrogel-type pharmaceutical formulations for wound treatment. Four alginate-based hydrogels (Alg, Alg_HA, Alg_S, and Alg_HA_S) were subsequently obtained and characterized from a physicochemical and biological point of view. 

As wound healing involves four steps, the hyaluronic acid-type component material in hydrogels manages certain processes during tissue recovery and is active in all four stages of healing. Moreover, hyaluronic acid can maintain a high moisture environment on the affected tissue [89]. Because rapid degradation and weak mechanical resistance are the limitations of hyaluronic acid in dressings applications, forming composite hydrogels with alginate is a strategy to overcome these impediments [90,91,92,93]. 

FTIR spectra recorded for all hydrogels supported the samples’ hydrophilic character by the appearance of a vibrational band at around 3200 cm^−1^. Alg_HA and Alg_HA_S seem to be the most hydrophilic materials due to the presence of the highest intense band for the OH group. All hydrogel spectra showed great similarity, except for the presence of C-H stretching at about 2907 cm^−1^ for only Alg_HA and Alg_HA_S, which is indicative of the confirmation of a hyaluronic alginate acid composite hydrogel [74,94]. Another clear observation is the Alg_HA spectrum, where the data show, at wavenumber 1520 cm^−1^, the NHCO group characteristic for hyaluronic acid. In the Alg_HA_S spectrum, the vibrational band was no longer evidenced after adding silver nanoparticles. This fact is probably due to the formation of the hyaluronic silver complex through the ionic bond of a carboxylic group with silver ions [95]. Taking into consideration the properties of an ideal wound dressing, it is necessary to optimize the synthesis process, especially to obtain a porous structure that plays multiple roles in the wound healing process (supports oxygen exchange at the injured site, enhances fibroblast production, and assures the absorption of the exudate). SEM analysis was performed on all hydrogels to investigate their morphostructures. The micrographs show the porous organization of the hydrogels, which results in one of the most important features of wound dressings. Alg and Alg_S had an average pore size of 56 µm and 65 µm, respectively, compared with Alg_HA and Alg_HA_S, where the average pore size was 63 µm and 59 µm, respectively. Lin et al. [96] made a composite wound dressing using alginate, hyaluronic acid, and collagen. An SEM investigation was also performed, facilitating the measurement of the pores, which indicated values of about 49 µm. Yang et al. [97] obtained silk fibroin, hyaluronic acid, and sodium alginate hydrogels with an average pore diameter of 93 µm. A hydrogel composed of polyvinyl alcohol, sodium alginate, and hyaluronic acid was synthesized by Jian et al. [98]. The pores were measured and ranged from 5 to 20 µm. In a study by Cantazano et al. [99], three formulations of hydrogels were obtained: alginate, alginate with 10% hyaluronic acid, and alginate with 20% hyaluronic acid. They observed that after the addition of hyaluronic acid, the size of the pores was increased, and the sample with 20% hyaluronic acid presented bigger pores compared with the samples with 10%. The swelling degree is a significant attribute regarding hydrogels’ wound dressing properties. The evaluation of Alg, Alg_S, Alg_HA, and Alg_HA_S was conducted in an SBF solution for 96 h. The first thing observed in the graphical results was the difference in the absorption capacity between the samples only with alginate and those with hyaluronic acid. Alginate-based hydrogels reached a maximum of 219% (Alg) and 196% (Alg_S) compared with the hydrogels with hyaluronic acid, where the maximum swelling degree was 126% (Alg_HA) and 102% (Alg_HA_S). In general, crosslinking of the hydrogels influenced the capacity for swelling. The higher the crosslinking, the lower the swelling degree, because the denser the hydrogel network, the lower the absorption capacity. Numerous studies have examined the influence of salts in hyaluronic acid crosslinking, and it was demonstrated that CaCl_2_ and MgCl_2_, for example, drastically decreased the swelling behavior of the hyaluronic acid-composed hydrogels [100,101,102,103]. The SBF testing solution played a role as a crosslinking agent in our swelling evaluation, and this is a possible explanation for why the hydrogels with hyaluronic acid presented a lower swelling capacity compared with only the alginate polymeric matrix. Yang et al. [97] developed a silk fibroin, hyaluronic acid, and sodium alginate scaffold with 42% swelling ratio results. Umar et al. [104] revealed the influence of hyaluronic acid in alginate hydrogels when the swelling degree was evaluated. They tested the hydrogels in a sodium chloride, calcium chloride, and phosphate buffer saline (PBS) solution. The amount of hyaluronic acid in the sample shows that the greater the amount of hyaluronic acid, the greater the ability to absorb liquid in the pores. The overall results indicate an increase in the swelling behavior when the hydrogels were in contact with PBS, compared with the other solution. The maximum swelling degree was 476% in PBS and 250% in the other solution.

The hydrogels were characterized using physicochemical techniques. The next step was to evaluate the fundamental criteria through which a wound dressing has efficiency against microorganisms due to the incorporation of some antimicrobial agents. In our study, the involvement of silver nanoparticles was assessed to have a concrete result in bacterial biofilm inhibition, in agreement with studies based on the silver bactericidal effect that have been developed and demonstrated in numerous research papers [105,106,107]. The infection of wounds can cause more than local physical injury to the host and may rapidly lead to a severe health condition. In addition, the contamination of wounds can be caused by Gram-positive or Gram-negative bacteria, or it can be more difficult to assess, as it can initially be caused by the opportunistic growth and colonization of bacteria usually found in the normal skin microbiome (e.g., *S. aureus*). Most studies demonstrate, after the first few weeks of infection, the localized bacteria are Gram-negative, such as *E. coli* [71,108,109,110]. In this sense, the antimicrobial evaluation of the hydrogels obtained in our study was performed on Gram-positive and Gram-negative strains at different point times (24, 48, and 72 h). The graphic representation of the log10 CFU/mL values (Figure 11) demonstrated the biofilm inhibition activity of the hydrogels with silver nanoparticles (Alg_S and Alg_HA_S). The most effective results were evaluated after 24 h when the Alg_HA_S hydrogel reached a significant decrease in CFu/mL value for both *S. aureus* and *E. coli* strains. In addition, the Alg_S sample promoted bactericidal activity, as observed after 24 h. The addition of hyaluronic acid in the hydrogel supported the slight difference between Alg_S and Alg_HA_S biofilm modulation by the bacteriostatic characteristic of the carbohydrate polymer. Even after 48 and 72 h, the biofilm inhibition activity of hydrogels was observed mostly on the *S. aureus* strain. The mechanism of silver nanoparticles has a guided action on the cell membrane of bacteria by collapsing it and leading to the end of the adenosine triphosphate (ATP) generation. This mechanism of silver nanoparticles has different behavior in Gram-positive and Gram-negative bacteria regarding bacterial cell membranes. In Gram-positive bacteria, the cell wall is represented by a single peptidoglycan layer that is thicker than the Gram-negative cell membrane, and it also has an outer membrane made of lipopolysaccharides. It was expected that the membrane of Gram-negative bacteria would be easier to collapse. Still, the positively charged silver ions were more strongly attracted to the negatively charged and thicker membrane of Gram-positive bacteria [71,107,111,112,113]. 

The biocompatibility of the materials in terms of skin dressing applications is a decisive parameter that can ensure optimal and proliferative interaction with the test cells. HaCaT cell viability was investigated in contact with the obtained hydrogels for 24 h. These first hours of treatment are the most important in terms of the wound healing process so as to ensure that all of the other stages an affected tissue goes through. The results indicate that all hydrogels were biocompatible compared with the control sample. The Alg sample seemed to induce the same behavior as the control (increased by 3% for Alg), and the other samples (Alg_HA, Alg_S, and ALg_HA_S) presented a proliferative activity that supported cell growth after 24 h of contact. Some studies revealed that hyaluronic acid supports the CD44 receptor in keratinocytes by managing their activities and epidermal purpose. In this sense, the hyaluronic acid in our hydrogels was connected with the CD44 receptor, leading to the migration and proliferation of keratinocytes that were in contact with them [114,115,116]. Specifically, for Alg_HA, an increase of 9% was recorded; for Alg_S, the viability was 13% higher, and for Alg_HA_S, a 10% difference was observed compared with the control. A study by O.Catanzano et al. [117] revealed the activity of alginate and alginate–hyaluronan composite hydrogels on the HaCaT cell line and supported the idea that these hydrogels could stimulate cell proliferation and were definitely biocompatible. Another study on HaCaT cell viability demonstrated that the interaction between this cell line and the membranes with alginate, hyaluronic acid, and silver nanoparticles suggested the biocompatibility of the materials [50]. R. Conte et al. [118] tested materials based on hyaluronic acid with resveratrol and chitosan nanoparticles from a cellular viability point of view. The assay was performed on the HaCaT cell line, and the results are in line with the expectations, demonstrating the suitable properties of the materials in contact with the keratinocytes.

Moreover, this interaction between the hyaluronic acid receptor (CD44) and keratinocytes also changed the cell shape and cytoskeleton by developing long protrusions termed lamellipodia. These changes were present in the fluorescence microscopy results for the hydrogels obtained in our study. Moreover, our results reveal that the fabricated hydrogels with silver nanoparticles (Alg_S and Alg_HA_S) had biocompatibility and a slightly proliferative character. 

According to our results and considering the interest in developing new materials enduring improved methods for wound treatment, the composite hydrogel based on alginate, hyaluronic acid, and silver nanoparticles may be a suitable and effective dressing. This hydrogel meets the basic criteria to be used in wound healing and to be considered as a valid solution. 

## 4. Materials and Methods

For the synthesis of silver nanoparticles and alginate–hyaluronic hydrogels, the following materials were purchased from Sigma-Aldrich and used in the study, silver nitrate (AgNO_3_), D-glucose (C_6_H_12_O_6_), sodium hydroxide (NaOH), and sodium alginate, which were of analytical purity and utilized without further purification. Hyaluronic acid (low molecular weight) was purchased from a local pharmacy. Distilled water was used throughout the synthesis process.

### 4.1. Microfluidic Platform Characteristics

The microfluidic platform was composed of poly(methyl methacrylate) (PMMA) and was obtained using the 1610 Pro laser cutting machine (RUBIQ CNC, Bacău, Romania). The schematic design was created using the RDWorksV8 8.01.54 software (Informer Technologies, Inc., Los Angeles, CA, USA). The microfluidic platform was composed of three plates with the same dimensional characteristics (length = 86 mm, width = 44 mm, and depth = 1 mm) [119].

A four-independent channel peristaltic pump with 12 rollers (Reglo ICC independent channel control pump) was used. The three inlet hoses—yellow three-stop color-coded tubing with an inner diameter of 1.52 mm—were used and unchanged during all of the syntheses.

### 4.2. Synthesis of Silver Nanoparticles

The silver nanoparticles were obtained by directly reducing the silver ions from the metallic precursor into a microfluidic platform, starting from 200 mL of 0.03 M AgNO_3_ aqueous solution and 400 mL of 0.25 M NaOH aqueous solution containing 1g of D-glucose. 

**Figure 14:**
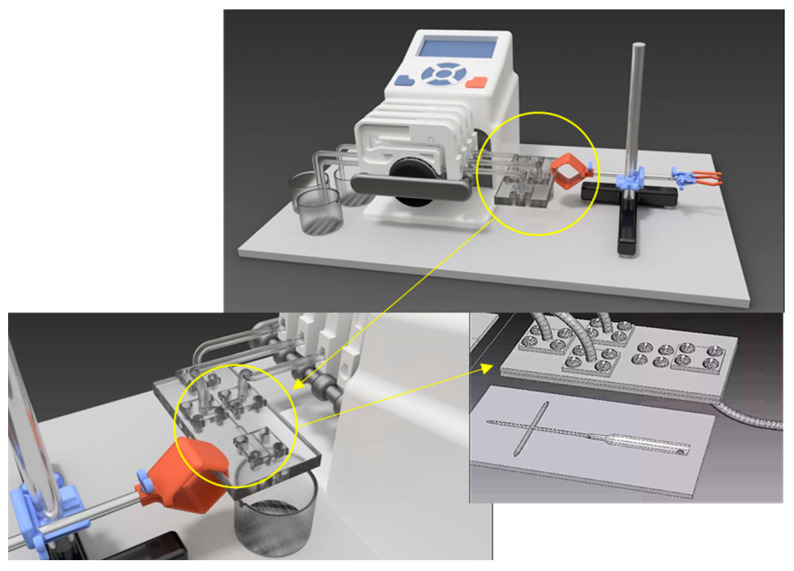
The schematic representation of the microfluidic synthesis of silver nanoparticles using a peristaltic pomp and a cross-shape microfluidic platform.

The design of the platform used was cross-shaped with three channel inputs in order to have an improved result in the reduction reaction. The metallic precursor solution entered the microfluidic platform using a central inlet, and the other two inputs were for the organic reduction solution. The experiment was controlled to use two concentrations of AgNO_3_ in the final reaction mixture, namely 0.03 M (series 1–0.5 wt.%) and 0.01 M (series 2–0.25 wt.%). For each series, the speed of the individual peristaltic pump channels (i.e., RPM) was varied, as presented in Table 4. A schematic representation of silver nanoparticles synthesis is detailed in Figure 14.

### 4.3. Synthesis of Alginate–Hyaluronic Acid-Based Hydrogels

Regarding the results from the physicochemical analysis obtained on silver nanoparticles, one sample, Ag(0.25_15), was chosen to form the antimicrobial and regenerative wound dressings. In order to obtain the hydrogels, a solution of 5% sodium alginate was prepared under continuous stirring for 1 h at room temperature, and the control sample containing only alginate was coded as Alg. Furthermore, the Alg_HA sample was obtained by mixing a 5% sodium alginate solution with a 3% hyaluronic acid solution (1:1 volume ratio). Both solutions were prepared under continuous stirring for 1 h at room temperature. Another sample (Alg_S) was formed by mixing a 5% sodium alginate solution (obtained similar to Alg sample process) with a silver nanoparticle powder sample of Ag(0.25_15) at a 2:1 (*v*:*w*) ratio. The mix was subjected to continuous stirring for 10 min at room temperature to distribute the silver nanoparticles through the polymer matrix. A hydrogel-coded Alg_HA_S was realized through sodium alginate and hyaluronic acid solutions (a solution of 5% alginate and 3% hyaluronic was prepared under magnetic stirring for 1 h at room environmental) with the addition of silver nanoparticle powder of Ag(0.25_15) at a 1:1:1 (*v*:*v*:*w*) ratio. The mix was left to stir for 10 min in order to have a homogenous silver distribution over the alginate–hyaluronic matrix. All of the hydrogels were placed in 5 cm diameter Petri dishes and left for 12 h in the freezer. After that, they were lyophilized at −80 °C for 72 h. The obtained materials were characterized by physicochemical analysis and evaluated on eukaryotic and prokaryotic cells. 

### 4.4. Characterization Techniques

#### 4.4.1. X-ray Diffraction (XRD)

The crystallinity and crystal parameter investigation of the silver nanoparticles was performed with an X-ray diffraction technique using a PANalytical Empyrean model diffractometer purchased from PANalytical, Almelo, The Netherlands), equipped with a hybrid monochromator (2xGe 220) on the incident side and a parallel plate collimator mounted on PIXcel 3D detector on the diffracted side. Grazing Incidence X-ray Diffraction (GIXRD) measurements were performed at room temperature, with an angle of incidence of ω = 0.5° for Bragg angle values of 2θ between 10° and 80°, using Cu Kα radiation with λ = 1.5406 Å (40 mA and 45 kV).

#### 4.4.2. Scanning Electron Microscopy (SEM)

To investigate the morphological information of the silver nanoparticles and alginate-based hydrogels, an SEM analysis was performed. The powder samples were fixed on a carbon-bearing slide and placed in an analysis chamber of an Inspect F50 scanning electron microscope. Hydrogels were also fixed on the carbon-bearing slide and covered with a thin gold film for 20 s, and then analyzed using the same parameters as the silver powder samples. The obtained images were created by recording the resulting secondary electron beam and electron beam scattering with an energy of 30 keV.

#### 4.4.3. Transmission Electron Microscopy (TEM)

A small amount of powder samples previously dispersed in water by ultrasonic treatment for 15 min was placed on a copper-coated copper grid and allowed to dry at room temperature. TEM images were obtained by analyzing the sample using a high-resolution 80–200 Titan Themis transmission electron type equipped with SAED, purchased from Thermofisher Scientific(Hillsboro, OR, USA). This microscope operates in transmission mode at a voltage of 200 kV, and the guaranteed point and line resolution have values of 2 Å and 1 Å, respectively.

#### 4.4.4. Dynamic Light Scattering (DLS)

DLS measurements were performed using a DelsaMax Pro-type device equipped with a 532 nm laser. The powders were dispersed in ultrapure water at room temperature. All samples were subjected to ultrasound for 10 min to achieve the best possible dispersion using an ultrasonic bath.

#### 4.4.5. Thermogravimetry-Differential Scanning Calorimetry (TG-DSC)

An STA 449C Jupiter device from Netzsch (NETZSCH-Gerätebau GmbH, Selb, Germany) was used for the thermal analysis (TG, DSC). Each sample was about 10 mg in weight. The samples were placed in an open alumina crucible and heated to 900 °C at a rate of 10 K/min, while being blown dried air at a rate of 50 mL/min. We utilized an empty alumina crucible as a point of reference. A thermostat gas-cell-equipped FTIR Tensor 27 from Bruker (Bruker Co., Ettlingen, Germany) was used to study the evolved gases.

#### 4.4.6. Fourier Transform Infrared Spectroscopy (FTIR)

For the identification of the functional compositional groups, FTIR analysis was performed. Using a ZnSe crystal of Thermo iN10-MX FTIR spectrometer purchased from Thermo Fisher Scientific, Waltham, USA, spectra collection was performed with measurements between 4000–400 cm^−1^.

#### 4.4.7. Swelling Rate

The lyophilized hydrogels were cut in a cylindrical shape with a 5 mm diameter to evaluate the swelling capacity of the obtained materials. Each sample was immersed in simulated body fluid (SBF) prepared according to Kokubo’s protocol in order to obtain a fluid similar to body plasma in terms of the ion content. The swelling rate was realized using the following formula:

$$Swelling\;ratio=\frac{Wt-Wi}{Wi}\times 100\%$$

where *Wi* is the initial mass of the sample (before SBF immersing), and *Wt* is the time point mass after immersing and weighing at different times. 

#### 4.4.8. Degradation Rate 

In addition, an important parameter of hydrogels is the degradation rate, referring more specifically to the incipient stage of degradation. In this sense, according to the formula below, the degradation rate was estimated.

(1) $$Degradation=\left(1-\frac{W0-Wt}{W0}\right)\times 100\%$$

#### 4.4.9. Antimicrobial Assessment—Biofilm Development

##### Bacterial Strains

Two clinically significant bacterial species, known as significant wound opportunistic pathogens, were used to test the antibacterial effectiveness of the alginate–hyaluronic acid-silver-based wound dressings in vitro. One Gram-positive (*Staphylococcus aureus* ATCC 25923) and one Gram-negative (*Escherichia coli* ATCC 25922) bacterial strain were used in this assay. The strains were from the culture collection of the Microbiology Immunology Department of the Faculty of Biology, University of Bucharest, as glycerol stocks at −80 °C.

##### Monospecific Biofilm Development

In this test, we assessed the effectiveness of the antimicrobial property of the dressings, both in the short- and long-term, against monospecific biofilms. The assay aimed to evaluate the formation of the bacterial biofilm in order to determine the antibiofilm effectiveness of the dressings. The materials for testing were cut into a disk with a 5 mm biopunch and sterilized by exposure to ultra-violet radiation for 20 min on each side. After the wound dressing samples were placed into sterile 24-well plates with 1 mL of nutritive broth, 10 μL of microbial suspension with a 0.5 McFarland standard density from each bacterial strain was added for inoculation. The next step was incubation for 24 h at 37 °C. After that, the culturing medium was removed, and 1 mL of sterile phosphate-buffered saline (PBS) was added for washing the dressings in order to discharge the unattached bacteria. A new sterile 24-well plate with fresh media was used to maintain the samples for 24, 48, and 72 h of incubation at 37 °C in order to achieve mature biofilm development. After every incubation, 1 mL of sterile phosphate-buffered saline solution was used to gently wash the wound dressings, which were then immersed in 1.5 mL centrifuge tubes containing 1000 μL PBS. The Eppendorf tubes were vortexed for about 30 s and subjected to ultrasound for 10 s to obtain the biofilm cell microbial suspensions. The obtained microbial suspensions were used to perform serial 10-fold dilutions inoculated on nutrient agar (solidified media) for obtaining and quantifying the colony-forming units (CFU/mL). 

##### Biocompatibility Assessment

Human keratinocytes (HaCaT cell line purchased from CLS Cell Lines Service GmbH, Eppelheim, Germany) were grown in Dulbecco Modified Eagle’s Medium (Invitrogen, Waltham, MA, USA) with 10% fetal bovine serum (Gibco, Grand Island, NY, USA) at 37 °C in a humidified atmosphere with 5% CO_2_. The cells were seeded at a cell density of 0.6 × 10^4^ cells/ cm^2^ and left overnight to adhere. Furthermore, the cells were incubated for 24 h with 1 mg/mL of ALG, ALG_HA, ALG_S, and ALG_HA_S prepared in cell culture media, which were previously sterilized under UV light for 3 h. In addition, the cells without any tested sample served as a negative control, and all of the results were expressed relative to this one. Moreover, a positive control represented by 1% Triton X-100 was performed in order to check whether all of the reagents were working properly, which obtained almost 100% cell death and LDH release.

##### MTT Assay

Cellular viability was assessed with 3-(4,5-dimethylthiazol-2-yl)-2,5-diphenyltetrazolium bromide (MTT; Sigma-Aldrich, St. Louis, MO, USA). The culture medium was removed, and the cells were incubated with 1 mg/mL MTT for 2 h at 37 °C. The purple formazan crystals formed in the viable cells were dissolved with 2-propanol (Sigma-Aldrich, St. Louis, MO, USA), and the absorbance was measured at 595 nm using a FlexStation 3 Benchtop Multi-Mode Microplate Reader (Molecular Devices, Sunnyvale, CA, USA).

##### Lactate Dehydrogenase (LDH) Release Measurement

LDH release was measured using Cytotoxicity Detection KitPLUS (Roche, Applied Science, Mannheim, Germany) according to the manufacturer’s instructions. Volumes of 100 µL culture supernatants collected after 24 h of keratinocyte growth in the presence of alginate-based hydrogels were mixed with a 100 µL reaction mixture made up of the catalyst and dye solution. After 30 min of incubation in a dark place, the absorbance was read at 490 nm using the Flex Station 3 microplate reader. 

##### F-actin Staining

At the end of incubation, the cells were fixed with 4% paraformaldehyde for 20 min and permeabilized with 0.1% Triton X−100−2% bovine serum albumin for 45 min. The actin filaments were stained with 10 µg/mL phalloidin–FITC (fluorescein isothiocyanate), and the nuclei were counterstained with 2 µg/mL DAPI (4′,6-diamino-2-phenylindole). The images were taken with an Olympus IX71 inverted fluorescence microscope (Olympus, Tokyo, Japan).

Statistical Analysis: The in vitro assays were performed in triplicate, and the results were presented as the mean ± standard deviation (SD) of three independent experiments. The statistical significance was analyzed by one-way analysis of variance (ANOVA) followed by a Bonferroni post hoc test for multiple comparisons using GraphPad Prism V. 9.0.0 software. A value of *p* less than 0.05 was considered significant [120].

## 5. Conclusions

Silver nanoparticles were synthesized through a microfluidic platform, using various mixing flow rates and precursor concentrations, in order to optimize the obtained nanoparticles in aspects of morphology, size, and dimensional distribution. Different physicochemical analyses were performed to evaluate the characteristics of the samples. The XRD data contributed to identifying the crystalline silver phase and calculating the crystallite size, confirming that reducing the flow of the organic solution resulted in an increase in the crystallite size of the samples. SEM analysis pointed out that the silver nitrate concentration influenced the morphology and size of the particles. Using a decreased silver nitrate concentration, the dimensional uniformity was maintained, and the size of the particles was smaller. TEM results support the information obtained from XRD data regarding the SAED pattern. The morphology was observed at a better resolution, highlighting the spherical and pseudo-spherical shapes. DLS measurements helped confirm the difference induced by the synthesis parameters, showing the smallest hydrodynamic radius in the Ag(0.25_15) sample. The optimal results for the aim of this study (to obtain small nanoparticles but with a low tendency of agglomeration and with a dimensional uniformity) were shown in the Ag(0.25_15) sample. 

In this sense, the chosen silver nanoparticles sample (Ag(0.25_15) was used to improve the wound dressing properties, as it is an antimicrobial agent that is an important aspect of wound healing. A desirable porous structure was observed in all alginate-based hydrogels with pore sizes in the range of 66–99 µm. It was observed that adding hyaluronic acid led to an increased hydrophilic feature of the hydrogels and improved the silver nanoparticle distribution through the hydrogel pores. A good swelling capacity was observed for all hydrogels during the evaluation time. Regarding the hydrogels’ antimicrobial activity, the biofilm modulation results demonstrated the well-known properties of silver nanoparticles as an effective antimicrobial agent. Alg_S and Alg_HA_S showed a high susceptibility to Gram-positive bacterial cells (*S. aureus*), with the best activity after 24 h and constant biofilm inhibition until 72 h. According to our results, adding hyaluronic acid to the hydrogels (Alg_HA_S) stimulated a decrease in CFU/mL compared with the hydrogel with only silver nanoparticles (Alg_S) due to the bacteriostatic activity of the carbohydrate polymer. Both bacterial strains of the biofilm (*S. aureus* and *E. coli*) showed significant inhibition in contact with the tested hydrogels (Alg_S and Alg_HA_S). The biological interaction between the obtained hydrogels and the selected keratinocyte cell line (HaCaT) strongly supports the required biocompatibility character of a wound dressing material. After 24 h of contact with the HaCat cells, the proliferative activity of the hydrogels with hyaluronic acid and silver nanoparticles was registered. 

To conclude, the obtained composite dressing, which combines the polymeric alginate matrix through the addition of hyaluronic acid, known for its properties in wound healing and its strength, and a biocompatible potent antimicrobial agent (i.e., silver nanoparticles), is a certain and future possibility for treating chronic and infected wounds. 

## Figures and Tables

**Table 1:** Crystallite size for the 0.5 wt.% sample series.

Sample	Ag(0.5_15all)	Ag(0.5_15)	Ag(0.5_30)	Ag(0.5_10)
Average crystallite size (nm)	47.415	45.0232	54.347	42.617

**Table 2:** Crystallite size for the 0.25 wt.% sample series.

Sample	Ag(0.25_15all)	Ag(0.25_15)	Ag(0.25_30)	Ag(0.25_10)
Average crystallite size (nm)	42.249	31.083	47.821	44.743

**Table 3:** Mass loss and thermic effect data registered for Alg, Alg_HA, Alg_S, and Alg_HA_S.

Sample	Mass Loss RT−210 °C	Mass Loss 210–280 °C	Mass Loss 280–400 °C	Effects Endo/Exo	Residual Mass	Ag %
Alg	14.10%	32.54%	9.04%	88.2/241.2 347.2/583.7	19.37%	-
Alg_HA	15.50%	34.44%	9.33%	85.2/243.9 331.9/596.0	13.63%	-
Alg_S	15.18%	32.33%	8.61%	89.1/242.0 346.0/602.5	20.39%	1.27%
Alg_HA_S	15.62%	34.80%	9.13%	82.8/245.3 325.7/608.7	13.94%	0.36%

**Table 4:** Experimental parameters and sample codes.

AgNO_3_ Channel	Reduction Solution Channels (×2)	Sample Code for 0.5 wt.% AgNO_3_	Sample Code for 0.25 wt.% AgNO_3_
15 RPM	15 RPM	Ag(0.5_15all)	Ag(0.25_15all)
15 RPM	30 RPM	Ag(0.5_15)	Ag(0.25_15)
30 RPM	15 RPM	Ag(0.5_30)	Ag(0.25_30)
10 RPM	15 RPM	Ag(0.5_10)	Ag(0.25_10)

## Data Availability

Not applicable.

## References

[1] Singh S.P., Bhargava C., Dubey V., Mishra A., Singh Y. (2017). Silver nanoparticles: Biomedical applications, toxicity, and safety issues. Int. J. Res. Pharm. Pharm. Sci..

[2] Gherasim O., Grumezescu A.M., Grumezescu V., Iordache F., Vasile B.S., Holban A.M. (2020). Bioactive surfaces of polylactide and silver nanoparticles for the prevention of microbial contamination. Materials.

[3] Spirescu V.A., Chircov C., Grumezescu A.M., Vasile B.Ș., Andronescu E. (2021). Inorganic nanoparticles and composite films for antimicrobial therapies. Int. J. Mol. Sci..

[4] Crisan C.M., Mocan T., Manolea M., Lasca L.I., Tăbăran F.-A., Mocan L. (2021). Review on silver nanoparticles as a novel class of antibacterial solutions. Appl. Sci..

[5] Karademir F., Ayhan F. (2021). Antimicrobial Surface Functionality of PEG Coated and AgNPs Immobilized Extracorporeal Biomaterials. Biointerface Res. Appl. Chem..

[6] Gounden S., Daniels A., Singh M. (2021). Chitosan-Modified Silver Nanoparticles Enhance Cisplatin Activity in Breast Cancer Cells. Biointerface Res. Appl. Chem..

[7] Lade B., Shanware A., Sharma R. (2022). Effect of β-Cyclodextrin Stabilized Silver Nanoparticles on Gills, Kidney, Liver of Danio rerio. Lett. Appl. NanoBioSci..

[8] Said A., Abu-Elghait M., Atta H.M., Salem S.S. (2023). Antibacterial Activity of Green Synthesized Silver Nanoparticles Using Lawsonia inermis Against Common Pathogens from Urinary Tract Infection. Appl. Biochem. Biotechnol..

[9] Salem S.S., Hashem A.H., Sallam A.-A.M., Doghish A.S., Al-Askar A.A., Arishi A.A., Shehabeldine A.M. (2022). Synthesis of Silver Nanocomposite Based on Carboxymethyl Cellulose: Antibacterial, Antifungal and Anticancer Activities. Polymers.

[10] Joy J., Gurumurthy M.S., Thomas R., Balachandran M. (2021). Biosynthesized Ag Nanoparticles: A Promising Pathway for Bandgap Tailoring. Biointerface Res. Appl. Chem..

[11] Lakshmanan M., Ragul D., Michael M.L., Moses J.A., Anandharamakrishanan C. (2022). Characterization of Silver Nanoparticles Synthesized Using *Ocimum basilicum* Seed Extract. Lett. Appl. NanoBioSci..

[12] Bruna T., Maldonado-Bravo F., Jara P., Caro N. (2021). Silver nanoparticles and their antibacterial applications. Int. J. Mol. Sci..

[13] Yaqoob A.A., Umar K., Ibrahim M.N.M. (2020). Silver nanoparticles: Various methods of synthesis, size affecting factors and their potential applications—A review. Appl. Nanosci..

[14] Xu L., Wang Y.-Y., Huang J., Chen C.-Y., Wang Z.-X., Xie H. (2020). Silver nanoparticles: Synthesis, medical applications and biosafety. Theranostics.

[15] Nakamura S., Sato M., Sato Y., Ando N., Takayama T., Fujita M., Ishihara M. (2019). Synthesis and application of silver nanoparticles (Ag NPs) for the prevention of infection in healthcare workers. Int. J. Mol. Sci..

[16] Cheon J.Y., Kim S.J., Rhee Y.H., Kwon O.H., Park W.H. (2019). Shape-dependent antimicrobial activities of silver nanoparticles. Int. J. Nanomed..

[17] Kaidi S., Belattmania Z., Bentiss F., Jama C., Reani A., Sabour B. (2021). Synthesis and characterization of silver nanoparticles using alginate from the brown seaweed *Laminaria ochroleuca*: Structural features and antibacterial activity. Biointerface Res. Appl. Chem..

[18] Lagashetty A., Ganiger S.K., Preeti R.K., Reddy S. (2021). Green Synthesis, Characterization and Antibacterial Study of Ag-Au Bimetallic Nanocomposite using Tea Powder Extract. Biointerface Res. Appl. Chem..

[19] Latha R., Sevarkodiyone S.P., Pandiarajan J. (2022). Multi-Faceted Role of Silver and Gold Nanoparticles Synthesized from Biowaste and its in vitro Antibacterial, Antifungal and Antidiabetic Activities. Lett. Appl. NanoBioSci..

[20] Elakraa A.A., Salem S.S., El-Sayyad G.S., Attia M.S. (2022). Cefotaxime incorporated bimetallic silver-selenium nanoparticles: Promising antimicrobial synergism, antibiofilm activity, and bacterial membrane leakage reaction mechanism. RSC Adv..

[21] Al-Rajhi A.M.H., Salem S.S., Alharbi A.A., Abdelghany T.M. (2022). Ecofriendly synthesis of silver nanoparticles using Kei-apple (*Dovyalis caffra*) fruit and their efficacy against cancer cells and clinical pathogenic microorganisms. Arab. J. Chem..

[22] Rodrigues A.G., Ruiz R.D., Selari P., de Araujo W.L., de Souza A.O. (2021). Anti-Biofilm Action of Biological Silver Nanoparticles Produced by *Aspergillus tubingensis* and Antimicrobial Activity of Fabrics Carrying it. Biointerface Res. Appl. Chem..

[23] Panda B.S. (2022). A Review on Synthesis of Silver Nanoparticles and their Biomedical Applications. Lett. Appl. NanoBioSci..

[24] Rai M., Ingle A.P., Trzcińska-Wencel J., Wypij M., Bonde S., Yadav A., Kratošová G., Golińska P. (2021). Biogenic silver nanoparticles: What we know and what do we need to know?. Nanomaterials.

[25] Kang K.-K., Lee B., Lee C.-S. (2019). Recent progress in the synthesis of inorganic particulate materials using microfluidics. J. Taiwan Inst. Chem. Eng..

[26] Nathanael K., Pico P., Kovalchuk N.M., Lavino A.D., Simmons M.J., Matar O.K. (2022). Computational modelling and microfluidics as emerging approaches to synthesis of silver nanoparticles–A review. Chem. Eng. J..

[27] Hou X., Zhang Y.S., Santiago G.T.-d., Alvarez M.M., Ribas J., Jonas S.J., Weiss P.S., Andrews A.M., Aizenberg J., Khademhosseini A. (2017). Interplay between materials and microfluidics. Nat. Rev. Mater..

[28] Fallahi H., Zhang J., Phan H.-P., Nguyen N.-T. (2019). Flexible microfluidics: Fundamentals, recent developments, and applications. Micromachines.

[29] Ma J., Wang Y., Liu J. (2017). Biomaterials meet microfluidics: From synthesis technologies to biological applications. Micromachines.

[30] Niculescu A.-G., Chircov C., Bîrcă A.C., Grumezescu A.M. (2021). Fabrication and applications of microfluidic devices: A review. Int. J. Mol. Sci..

[31] Hung L.-H., Lee A.P. (2007). Microfluidic devices for the synthesis of nanoparticles and biomaterials. J. Med. Biol. Eng..

[32] Kung C.-T., Gao H., Lee C.-Y., Wang Y.-N., Dong W., Ko C.-H., Wang G., Fu L.-M. (2020). Microfluidic synthesis control technology and its application in drug delivery, bioimaging, biosensing, environmental analysis and cell analysis. Chem. Eng. J..

[33] Ma J., Lee S.M.-Y., Yi C., Li C.-W. (2017). Controllable synthesis of functional nanoparticles by microfluidic platforms for biomedical applications—A review. Lab A Chip.

[34] Diniz F.R., Maia R.C.A., de Andrade L.R.M., Andrade L.N., Vinicius Chaud M., da Silva C.F., Corrêa C.B., de Albuquerque Junior R.L.C., Pereira da Costa L., Shin S.R. (2020). Silver nanoparticles-composing alginate/gelatine hydrogel improves wound healing in vivo. Nanomaterials.

[35] Nqakala Z.B., Sibuyi N.R., Fadaka A.O., Meyer M., Onani M.O., Madiehe A.M. (2021). Advances in nanotechnology towards development of silver nanoparticle-based wound-healing agents. Int. J. Mol. Sci..

[36] Shehabeldine A.M., Salem S.S., Ali O.M., Abd-Elsalam K.A., Elkady F.M., Hashem A.H. (2022). Multifunctional silver nanoparticles based on chitosan: Antibacterial, antibiofilm, antifungal, antioxidant, and wound-healing activities. J. Fungi.

[37] Mehwish H.M., Liu G., Rajoka M.S.R., Cai H., Zhong J., Song X., Xia L., Wang M., Aadil R.M., Inam-Ur-Raheem M. (2021). Therapeutic potential of *Moringa oleifera* seed polysaccharide embedded silver nanoparticles in wound healing. Int. J. Biol. Macromol..

[38] Zhang K., Lui V.C., Chen Y., Lok C.N., Wong K.K. (2020). Delayed application of silver nanoparticles reveals the role of early inflammation in burn wound healing. Sci. Rep..

[39] Bhubhanil S., Talodthaisong C., Khongkow M., Namdee K., Wongchitrat P., Yingmema W., Hutchison J.A., Lapmanee S., Kulchat S. (2021). Enhanced wound healing properties of guar gum/curcumin-stabilized silver nanoparticle hydrogels. Sci. Rep..

[40] Kuznetsova T.A., Andryukov B.G., Besednova N.N., Zaporozhets T.S., Kalinin A.V. (2020). Marine algae polysaccharides as basis for wound dressings, drug delivery, and tissue engineering: A review. J. Mar. Sci. Eng..

[41] Aderibigbe B.A., Buyana B. (2018). Alginate in wound dressings. Pharmaceutics.

[42] Varaprasad K., Jayaramudu T., Kanikireddy V., Toro C., Sadiku E.R. (2020). Alginate-based composite materials for wound dressing application: A mini review. Carbohydr. Polym..

[43] Sikka M.P., Midha V.K. (2019). The role of biopolymers and biodegradable polymeric dressings in managing chronic wounds. Advanced Textiles for Wound Care.

[44] Xie Y., Gao P., He F., Zhang C. (2022). Application of alginate-based hydrogels in hemostasis. Gels.

[45] Zhang M., Zhao X. (2020). Alginate hydrogel dressings for advanced wound management. Int. J. Biol. Macromol..

[46] Johnson K.-a., Muzzin N., Toufanian S., Slick R.A., Lawlor M.W., Seifried B., Moquin P., Latulippe D., Hoare T. (2020). Drug-impregnated, pressurized gas expanded liquid-processed alginate hydrogel scaffolds for accelerated burn wound healing. Acta Biomater..

[47] Wang T., Zheng Y., Shi Y., Zhao L. (2019). pH-responsive calcium alginate hydrogel laden with protamine nanoparticles and hyaluronan oligosaccharide promotes diabetic wound healing by enhancing angiogenesis and antibacterial activity. Drug Deliv. Transl. Res..

[48] Rymar S., Pikus P., Buchek P., Shuvalova N., Pokholenko I., Irodov D., Kordium V. (2022). Comparison of the Therapeutic Effects of hUC-MSC Intravenous Delivery and Intraperitoneal Administration of MSCs Encapsulated in Alginate Capsules for the Treatment of Rat Liver Cirrhosis. Biointerface Res. Appl. Chem..

[49] Kushwaha S., Pandey A.K., Kushwaha N. (2022). Role of Nanomaterials in Cosmeceuticals: Challenges, Development Strategies, and Future Perspective. Lett. Appl. NanoBioSci..

[50] Tarusha L., Paoletti S., Travan A., Marsich E. (2018). Alginate membranes loaded with hyaluronic acid and silver nanoparticles to foster tissue healing and to control bacterial contamination of non-healing wounds. J. Mater. Sci. Mater. Med..

[51] Chalitangkoon J., Wongkittisin M., Monvisade P. (2020). Silver loaded hydroxyethylacryl chitosan/sodium alginate hydrogel films for controlled drug release wound dressings. Int. J. Biol. Macromol..

[52] Kumar S.S.D., Rajendran N.K., Houreld N.N., Abrahamse H. (2018). Recent advances on silver nanoparticle and biopolymer-based biomaterials for wound healing applications. Int. J. Biol. Macromol..

[53] Choudhary M., Chhabra P., Tyagi A., Singh H. (2021). Scar free healing of full thickness diabetic wounds: A unique combination of silver nanoparticles as antimicrobial agent, calcium alginate nanoparticles as hemostatic agent, fresh blood as nutrient/growth factor supplier and chitosan as base matrix. Int. J. Biol. Macromol..

[54] Kaczmarek-Pawelska A. (2019). Alginate-based hydrogels in regenerative medicine. Alginates: Recent Uses of This Natural Polymer.

[55] Srichaiyapol O., Maddocks S.E., Thammawithan S., Daduang S., Klaynongsruang S., Patramanon R. (2022). TA-AgNPs/Alginate Hydrogel and Its Potential Application as a Promising Antibiofilm Material against Polymicrobial Wound Biofilms Using a Unique Biofilm Flow Model. Microorganisms.

[56] Fang Y., Shi L., Duan Z., Rohani S. (2021). Hyaluronic acid hydrogels, as a biological macromolecule-based platform for stem cells delivery and their fate control: A review. Int. J. Biol. Macromol..

[57] Zamboni F., Wong C.K., Collins M.N. (2023). Hyaluronic acid association with bacterial, fungal and viral infections: Can hyaluronic acid be used as an antimicrobial polymer for biomedical and pharmaceutical applications?. Bioact. Mater..

[58] Zamboni F., Vieira S., Reis R.L., Oliveira J.M., Collins M.N. (2018). The potential of hyaluronic acid in immunoprotection and immunomodulation: Chemistry, processing and function. Prog. Mater. Sci..

[59] Della Sala F., Longobardo G., Fabozzi A., di Gennaro M., Borzacchiello A. (2022). Hyaluronic acid-based wound dressing with antimicrobial properties for wound healing application. Appl. Sci..

[60] Ding Y.-W., Zhang X.-W., Mi C.-H., Qi X.-Y., Zhou J., Wei D.-X. (2022). Recent advances in hyaluronic acid-based hydrogels for 3D bioprinting in tissue engineering applications. Smart Mater. Med..

[61] Bertuola M., Aráoz B., Gilabert U., Gonzalez-Wusener A., Pérez-Recalde M., Arregui C.O., Hermida É.B. (2021). Gelatin–alginate–hyaluronic acid inks for 3D printing: Effects of bioglass addition on printability, rheology and scaffold tensile modulus. J. Mater. Sci..

[62] Prasathkumar M., Sadhasivam S. (2021). Chitosan/Hyaluronic acid/Alginate and an assorted polymers loaded with honey, plant, and marine compounds for progressive wound healing—Know-how. Int. J. Biol. Macromol..

[63] Rossi S., Mori M., Vigani B., Bonferoni M., Sandri G., Riva F., Caramella C., Ferrari F. (2018). A novel dressing for the combined delivery of platelet lysate and vancomycin hydrochloride to chronic skin ulcers: Hyaluronic acid particles in alginate matrices. Eur. J. Pharm. Sci..

[64] Mahmood U., Masood R., Afzal M.A., Raza Z.A., Abid S., Zahir A., Hussain T., Nazir A. (2022). Development of zinc, silver, and hyaluronic acid mediated wet spun alginate fibers for potential wound care applications. J. Ind. Text..

[65] Graça M.F., Miguel S.P., Cabral C.S., Correia I.J. (2020). Hyaluronic acid—Based wound dressings: A review. Carbohydr. Polym..

[66] Silvestro I., Lopreiato M., Scotto d’Abusco A., Di Lisio V., Martinelli A., Piozzi A., Francolini I. (2020). Hyaluronic acid reduces bacterial fouling and promotes fibroblasts’ adhesion onto chitosan 2D-wound dressings. Int. J. Mol. Sci..

[67] Saitarly S., Pushkarev Y., Nesterkina M., Ozturk S., Salih B., Kravchenko I. (2022). Rheological Properties of Hyaluronic Acid Diluted Solutions as Components of Cosmetics. Biointerface Res. Appl. Chem..

[68] Kaya G., Oytun F. (2021). Rheological Properties of Injectable Hyaluronic Acid Hydrogels for Soft Tissue Engineering Applications. Biointerface Res. Appl. Chem..

[69] Prakash P., Sowmya N., Sudha S., Masilamani Selvam M., Pully D., Shobana N., Saigeetha S., Samrot A.V. (2022). Isolation and Characterization of Chitin Nanofibers from *Calocybe indica* and its Applications. Lett. Appl. NanoBioSci..

[70] Abou-Okeil A., Fahmy H.M., El-Bisi M.K., Ahmed-Farid O.A. (2018). Hyaluronic acid/Na-alginate films as topical bioactive wound dressings. Eur. Polym. J..

[71] Catanzano O., D’Esposito V., Pulcrano G., Maiolino S., Ambrosio M.R., Esposito M., Miro A., Ungaro F., Formisano P., Catania M.R. (2017). Ultrasmall silver nanoparticles loaded in alginate–hyaluronic acid hybrid hydrogels for treating infected wounds. Int. J. Polym. Mater. Polym. Biomater..

[72] Serafin A., Culebras M., Collins M.N. (2023). Synthesis and evaluation of alginate, gelatin, and hyaluronic acid hybrid hydrogels for tissue engineering applications. Int. J. Biol. Macromol..

[73] Jiang B.-P., Zhang L., Zhu Y., Shen X.-C., Ji S.-C., Tan X.-Y., Cheng L., Liang H. (2015). Water-soluble hyaluronic acid–hybridized polyaniline nanoparticles for effectively targeted photothermal therapy. J. Mater. Chem. B.

[74] Fischer R.L., McCoy M.G., Grant S.A. (2012). Electrospinning collagen and hyaluronic acid nanofiber meshes. J. Mater. Sci. Mater. Med..

[75] Salem S.S. (2023). A mini review on green nanotechnology and its development in biological effects. Arch. Microbiol..

[76] Salem S.S., Fouda A. (2021). Green Synthesis of Metallic Nanoparticles and Their Prospective Biotechnological Applications: An Overview. Biol. Trace Elem. Res..

[77] Xu L., Peng J., Yan M., Zhang D., Shen A.Q. (2016). Droplet synthesis of silver nanoparticles by a microfluidic device. Chem. Eng. Process. Process Intensif..

[78] Siddiqi K.S., Husen A., Rao R.A. (2018). A review on biosynthesis of silver nanoparticles and their biocidal properties. J. Nanobiotechnol..

[79] Calderón-Jiménez B., Johnson M.E., Montoro Bustos A.R., Murphy K.E., Winchester M.R., Vega Baudrit J.R. (2017). Silver nanoparticles: Technological advances, societal impacts, and metrological challenges. Front. Chem..

[80] Hong T., Lu A., Liu W., Chen C. (2019). Microdroplet synthesis of silver nanoparticles with controlled sizes. Micromachines.

[81] Sobczak-Kupiec A., Malina D., Wzorek Z., Zimowska M. (2011). Influence of silver nitrate concentration on the properties of silver nanoparticles. Micro Nano Lett..

[82] Demchenko V., Riabov S., Kobylinskyi S., Goncharenko L., Rybalchenko N., Kruk A., Moskalenko O., Shut M. (2020). Effect of the type of reducing agents of silver ions in interpolyelectrolyte-metal complexes on the structure, morphology and properties of silver-containing nanocomposites. Sci. Rep..

[83] Ng C.M., Chen P.C., Manickam S. (2012). Green high-gravitational synthesis of silver nanoparticles using a rotating packed bed reactor (RPBR). Ind. Eng. Chem. Res..

[84] Lazarus L.L., Riche C.T., Marin B.C., Gupta M., Malmstadt N., Brutchey R.L. (2012). Two-phase microfluidic droplet flows of ionic liquids for the synthesis of gold and silver nanoparticles. ACS Appl. Mater. Interfaces.

[85] Baber R., Mazzei L., Thanh N.T., Gavriilidis A. (2015). Synthesis of silver nanoparticles in a microfluidic coaxial flow reactor. RSC Adv..

[86] Kim T.H., Kim M., Park H.S., Shin U.S., Gong M.S., Kim H.W. (2012). Size-dependent cellular toxicity of silver nanoparticles. J. Biomed. Mater. Res. Part A.

[87] Liu W., Wu Y., Wang C., Li H.C., Wang T., Liao C.Y., Cui L., Zhou Q.F., Yan B., Jiang G.B. (2010). Impact of silver nanoparticles on human cells: Effect of particle size. Nanotoxicology.

[88] Magdalene D.J., Muthuselvam D., Pravinraj T. (2021). Microfluidics-based green synthesis of silver nanoparticle from the aqueous leaf extract of *Ipomea quamoclit* L.. Appl. Nanosci..

[89] Cortes H., Caballero-Florán I.H., Mendoza-Muñoz N., Córdova-Villanueva E.N., Escutia-Guadarrama L., Figueroa-González G., Reyes-Hernández O.D., González-Del Carmen M., Varela-Cardoso M., Magaña J.J. (2020). Hyaluronic acid in wound dressings. Cell. Mol. Biol..

[90] Thanh T.N., Laowattanatham N., Ratanavaraporn J., Sereemaspun A., Yodmuang S. (2022). Hyaluronic acid crosslinked with alginate hydrogel: A versatile and biocompatible bioink platform for tissue engineering. Eur. Polym. J..

[91] Jamalzaei P., Valojerdi M.R., Montazeri L., Baharvand H. (2020). Applicability of hyaluronic acid-alginate hydrogel and ovarian cells for in vitro development of mouse preantral follicles. Cell J..

[92] Hasan N., Cao J., Lee J., Kim H., Yoo J.-W. (2021). Development of clindamycin-loaded alginate/pectin/hyaluronic acid composite hydrogel film for the treatment of MRSA-infected wounds. J. Pharm. Investig..

[93] Pérez-Madrigal M.M., Shaw J.E., Arno M.C., Hoyland J.A., Richardson S.M., Dove A.P. (2020). Robust alginate/hyaluronic acid thiol–yne click-hydrogel scaffolds with superior mechanical performance and stability for load-bearing soft tissue engineering. Biomater. Sci..

[94] Godoy-Alvarez F.K., González-Torres M., Giraldo-Gomez D.M., Sánchez-Sánchez R., Pérez-Díaz M.A., González-Del Carmen M., Figueroa-González G., Reyes-Hernández O.D., Sharifi-Rad J., Cortés H. (2021). Synthesis by gamma irradiation of hyaluronic acid-polyvinyl alcohol hydrogel for biomedical applications. Cell. Mol. Biol..

[95] Chudobova D., Nejdl L., Gumulec J., Krystofova O., Rodrigo M.A.M., Kynicky J., Ruttkay-Nedecky B., Kopel P., Babula P., Adam V. (2013). Complexes of silver (I) ions and silver phosphate nanoparticles with hyaluronic acid and/or chitosan as promising antimicrobial agents for vascular grafts. Int. J. Mol. Sci..

[96] Lin Z., Wu T., Wang W., Li B., Wang M., Chen L., Xia H., Zhang T. (2019). Biofunctions of antimicrobial peptide-conjugated alginate/hyaluronic acid/collagen wound dressings promote wound healing of a mixed-bacteria-infected wound. Int. J. Biol. Macromol..

[97] Yang W., Xu H., Lan Y., Zhu Q., Liu Y., Huang S., Shi S., Hancharou A., Tang B., Guo R. (2019). Preparation and characterisation of a novel silk fibroin/hyaluronic acid/sodium alginate scaffold for skin repair. Int. J. Biol. Macromol..

[98] Jiang Y., Hou Y., Fang J., Liu W., Zhao Y., Huang T., Cui J., Yang Y., Zhou Z. (2019). Preparation and characterization of PVA/SA/HA composite hydrogels for wound dressing. Int. J. Polym. Anal. Charact..

[99] Catanzano O., D′Esposito V., Formisano P., Boateng J.S., Quaglia F. (2018). Composite alginate-hyaluronan sponges for the delivery of tranexamic acid in postextractive alveolar wounds. J. Pharm. Sci..

[100] Wang S., Nawale G.N., Oommen O.P., Hilborn J., Varghese O.P. (2019). Influence of ions to modulate hydrazone and oxime reaction kinetics to obtain dynamically cross-linked hyaluronic acid hydrogels. Polym. Chem..

[101] Salma-Ancane K., Sceglovs A., Tracuma E., Wychowaniec J.K., Aunina K., Ramata-Stunda A., Nikolajeva V., Loca D. (2022). Effect of crosslinking strategy on the biological, antibacterial and physicochemical performance of hyaluronic acid and ɛ-polylysine based hydrogels. Int. J. Biol. Macromol..

[102] Xue Y., Chen H., Xu C., Yu D., Xu H., Hu Y. (2020). Synthesis of hyaluronic acid hydrogels by crosslinking the mixture of high-molecular-weight hyaluronic acid and low-molecular-weight hyaluronic acid with 1, 4-butanediol diglycidyl ether. RSC Adv..

[103] Schanté C.E., Zuber G., Herlin C., Vandamme T.F. (2011). Chemical modifications of hyaluronic acid for the synthesis of derivatives for a broad range of biomedical applications. Carbohydr. Polym..

[104] Umar M., Ullah A., Nawaz H., Areeb T., Hashmi M., Kharaghani D., Kim K.O., Kim I.S. (2021). Wet-spun bi-component alginate based hydrogel fibers: Development and in-vitro evaluation as a potential moist wound care dressing. Int. J. Biol. Macromol..

[105] Hassanien A.S., Khatoon U.T. (2019). Synthesis and characterization of stable silver nanoparticles, Ag-NPs: Discussion on the applications of Ag-NPs as antimicrobial agents. Phys. B Condens. Matter.

[106] Das C.A., Kumar V.G., Dhas T.S., Karthick V., Govindaraju K., Joselin J.M., Baalamurugan J. (2020). Antibacterial activity of silver nanoparticles (biosynthesis): A short review on recent advances. Biocatal. Agric. Biotechnol..

[107] Yin I.X., Zhang J., Zhao I.S., Mei M.L., Li Q., Chu C.H. (2020). The antibacterial mechanism of silver nanoparticles and its application in dentistry. Int. J. Nanomed..

[108] Valentine K.P., Viacheslav K.M. (2017). Bacterial flora of combat wounds from eastern Ukraine and time-specified changes of bacterial recovery during treatment in Ukrainian military hospital. BMC Res. Notes.

[109] Taneja N., Chari P., Singh M., Singh G., Biswal M., Sharma M. (2013). Evolution of bacterial flora in burn wounds: Key role of environmental disinfection in control of infection. Int. J. Burn. Trauma.

[110] Raz-Pasteur A., Hussein K., Finkelstein R., Ullmann Y., Egozi D. (2013). Blood stream infections (BSI) in severe burn patients—Early and late BSI: A 9-year study. Burns.

[111] Mohsen E., El-Borady O.M., Mohamed M.B., Fahim I.S. (2020). Synthesis and characterization of ciprofloxacin loaded silver nanoparticles and investigation of their antibacterial effect. J. Radiat. Res. Appl. Sci..

[112] Alsamhary K.I. (2020). Eco-friendly synthesis of silver nanoparticles by Bacillus subtilis and their antibacterial activity. Saudi J. Biol. Sci..

[113] Mohammed A.M., Hassan K.T., Hassan O.M. (2023). Assessment of antimicrobial activity of chitosan/silver nanoparticles hydrogel and cryogel microspheres. Int. J. Biol. Macromol..

[114] Bourguignon L.Y. (2014). Matrix hyaluronan-activated CD44 signaling promotes keratinocyte activities and improves abnormal epidermal functions. Am. J. Pathol..

[115] Karvinen S., Pasonen-Seppänen S., Hyttinen J.M., Pienimäki J.-P., Törrönen K., Jokela T.A., Tammi M.I., Tammi R. (2003). Keratinocyte growth factor stimulates migration and hyaluronan synthesis in the epidermis by activation of keratinocyte hyaluronan synthases 2 and 3. J. Biol. Chem..

[116] Hartmann-Petersen S. (2009). Hyaluronan and CD44 in Epidermis with Special Reference to Growth Factors and Malignant Transformation (Kasvutekijöiden ja Syövän Vaikutus Epidermiksen Hyaluronaaniin ja CD44: Ään). Ph.D. Thesis.

[117] Catanzano O., D’esposito V., Acierno S., Ambrosio M., De Caro C., Avagliano C., Russo P., Russo R., Miro A., Ungaro F. (2015). Alginate–hyaluronan composite hydrogels accelerate wound healing process. Carbohydr. Polym..

[118] Conte R., De Luca I., Valentino A., Cerruti P., Pedram P., Cabrera-Barjas G., Moeini A., Calarco A. (2023). Hyaluronic Acid Hydrogel Containing Resveratrol-Loaded Chitosan Nanoparticles as an Adjuvant in Atopic Dermatitis Treatment. J. Funct. Biomater..

[119] Chircov C., Bîrcă A.C., Grumezescu A.M., Vasile B.S., Oprea O., Nicoară A.I., Yang C.-H., Huang K.-S., Andronescu E. (2021). Synthesis of magnetite nanoparticles through a lab-on-chip device. Materials.

[120] Florea D.A., Grumezescu V., Bîrcă A.C., Vasile B.Ș., Iosif A., Chircov C., Stan M.S., Grumezescu A.M., Andronescu E., Chifiriuc M.C. (2022). Bioactive Hydroxyapatite-Magnesium Phosphate Coatings Deposited by MAPLE for Preventing Infection and Promoting Orthopedic Implants Osteointegration. Materials.

